# Genomic signatures of convergent adaptation to Alpine environments in three Brassicaceae species

**DOI:** 10.1111/mec.15648

**Published:** 2020-10-14

**Authors:** Christian Rellstab, Stefan Zoller, Christian Sailer, Andrew Tedder, Felix Gugerli, Kentaro K. Shimizu, Rolf Holderegger, Alex Widmer, Martin C. Fischer

**Affiliations:** ^1^ Swiss Federal Research Institute WSL Birmensdorf Switzerland; ^2^ Genetic Diversity Centre (GDC) ETH Zurich Zurich Switzerland; ^3^ Institute of Integrative Biology (IBZ) ETH Zurich Zurich Switzerland; ^4^ Department of Evolutionary Biology and Environmental Studies Department of Plant and Microbial Biology University of Zurich Zurich Switzerland; ^5^ School of Chemistry & Bioscience University of Bradford Bradford UK; ^6^ Kihara Institute for Biological Research Yokohama City University Yokohama Japan

**Keywords:** adaptation, Alpine environment, Brassicaceae, environmental association, genome assembly, genome scans

## Abstract

It has long been discussed to what extent related species develop similar genetic mechanisms to adapt to similar environments. Most studies documenting such convergence have either used different lineages within species or surveyed only a limited portion of the genome. Here, we investigated whether similar or different sets of orthologous genes were involved in genetic adaptation of natural populations of three related plant species to similar environmental gradients in the Alps. We used whole‐genome pooled population sequencing to study genome‐wide SNP variation in 18 natural populations of three Brassicaceae (*Arabis alpina, Arabidopsis halleri,* and *Cardamine resedifolia*) from the Swiss Alps. We first de novo assembled draft reference genomes for all three species. We then ran population and landscape genomic analyses with ~3 million SNPs per species to look for shared genomic signatures of selection and adaptation in response to similar environmental gradients acting on these species. Genes with a signature of convergent adaptation were found at significantly higher numbers than expected by chance. The most closely related species pair showed the highest relative over‐representation of shared adaptation signatures. Moreover, the identified genes of convergent adaptation were enriched for nonsynonymous mutations, suggesting functional relevance of these genes, even though many of the identified candidate genes have hitherto unknown or poorly described functions based on comparison with *Arabidopsis thaliana*. We conclude that adaptation to heterogeneous Alpine environments in related species is partly driven by convergent evolution, but that most of the genomic signatures of adaptation remain species‐specific.

## 1 INTRODUCTION

Evolutionary biologists have long been fascinated by convergent adaptation to environmental conditions and the underlying genetic mechanisms (Endler, 1986; Losos, 2011; Stern, 2013). A classic example of convergent climatic adaptation was established by Clausen et al. (1940), who demonstrated that several species in the plant genera *Achillea*, *Artemisia*, and *Potentilla* have evolved alpine and lowland ecotypes/species that show similar phenotypic variants in response to altitude. From the genetic perspective, convergent adaptation based on the same genetic mechanisms can have two different origins (Stern, 2013). Novel mutations leading to the same beneficial phenotype can occur independently in different lineages, for example the development of C4 photosynthesis in grasses by independent mutations in similar or identical key amino acids (Christin et al., 2007) and the evolution of self‐compatibility in Brassicaceae species by independent loss‐of‐function mutations of the *SCR/SP11* gene (Shimizu & Tsuchimatsu, 2015). Alternatively, lineages share beneficial alleles that originate from standing genetic variation. These alleles have their origin either in a shared ancestral population, for example in the repeated adaptation of sticklebacks to freshwater environments through the same alleles in the *ectodysplasin* gene (Colosimo et al., 2005; Jones et al., 2012), or they are derived from introgression from a hybridizing species, exemplified by wing colour patterns in *Heliconius* butterflies (Dasmahapatra et al., 2012).

Historically, convergent adaptation has been mainly identified in studies of experimental evolution, phylogenetics, and quantitative genetics (Arendt & Reznick, 2008; Wood et al., 2005). Recently, genomic analyses in natural populations have also uncovered patterns of convergent adaptation (for examples see Stern, 2013). Advances in next‐generation sequencing (NGS) technologies allow one to searching for signatures of selection in whole genomes of natural populations (Savolainen et al., 2013; Stapley et al., 2010; Weigel & Nordborg, 2015). In combination with environmental data of high spatial resolution, this enables accurate inference of signatures of adaptation to climate through environmental association analysis (EAA, Rellstab et al., 2015). By comparing signatures of adaptation across related species living along similar environmental gradients, one is able to assess the relative levels of convergent or nonconvergent genetic adaptation. However, comparative studies of population and landscape genomics across species have been rare so far (but see e.g., Bedford & Hoekstra, 2015; Yeaman et al., 2016; Zhao & Begun, 2017; Zhao et al., 2015). These studies have revealed first insight into convergent adaptation at the genomic level. For example, Yeaman et al. (2016) showed that two conifer species, which separated approx. 140 million years ago, share a substantial amount of genomic signatures of adaptation. Similarly, Zhao and Begun (2017) revealed that similar gene sets in two *Drosophila* species, which diverged around 50 million years ago, showed signatures of adaptation in relation to the colonization of high latitudes.

A large proportion of genomic studies investigating adaptation focuses on terrestrial plant species (Ahrens et al., 2018). Plants as largely immobile organisms are well suited for studying environmental adaptation, as they need to cope with their local environment without much opportunity for altering their site, enabling their observation in their natural habitats (Weigel & Nordborg, 2015). However, identifying signatures of selection as a consequence of adaptation in natural populations requires strong drivers of selection, which are most efficiently detected in heterogeneous environments over small spatial scales, i.e. within gene flow distance (Lotterhos & Whitlock, 2015; Tigano & Friesen, 2016). Alpine environments are ideal for such studies, as they exhibit strong environmental variation over short geographic distances as a consequence of rugged topography and short‐distance variation in altitude.

**FIGURE 1:**
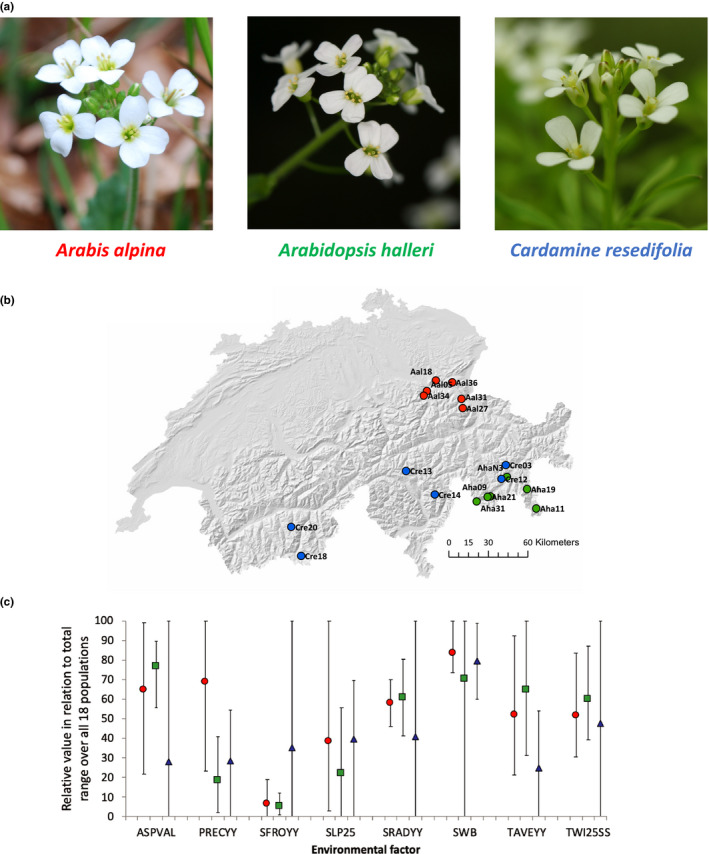
Study system. (a) The three study species from the Brassicaceae family. From left to right: flowers of *Arabis alpina* (Aal, red), *Arabidopsis halleri* (Aha, green), and *Cardamine resedifolia* (Cre, blue). (b) Sampling locations of the 18 populations of the three studied species in Switzerland. (c) Ecological overlap among the populations of the three species for the eight studied environmental factors. Given is the relative average and range (in respect to the total range over all 18 populations) of all three species. ASPVAL, aspect; PRECYY, yearly precipitation; SFROYY, yearly frost days; SLP25, slope; SRADYY, yearly solar radiation; SWB, site water balance; TAVEYY, yearly temperature; TWI25SS, topographic wetness index. For more details on environmental factors see Table S1. Photos by C. Rellstab (left) and M. C. Fischer (middle and right)

In this study, we were interested in the extent of genetic adaptation to similar environmental gradients in natural populations of three related plant species from the Brassicaceae family, namely *Arabis alpina*, *Arabidopsis halleri*, and *Cardamine resedifolia* (Figure 1a), based on variation at the same genes. The three study species are biologically and genetically rather divergent (Clauss & Koch, 2006). The genera *Arabis* and *Arabidopsis* diverged approximately 23 million years ago (Hohmann et al., 2015), while the divergence time between the more closely related genera *Cardamine* and *Arabidopsis* has been estimated to be at least 13 million years (Beilstein et al., 2010; Couvreur et al., 2010). We used pooled population sequencing (Pool‐Seq; Rellstab et al., 2013; Schlötterer et al., 2014) to study genome‐wide patterns of single‐nucleotide polymorphisms (SNPs) in natural populations of the three study species in the Swiss Alps (Figure 1b). We established de novo assembled draft genomes and ran population and landscape genomic analyses at the genome‐wide level to look for shared signatures of adaptation to similar environmental gradients (Figure 1c) across species. This study concentrated on shared signatures of environment‐driven adaptation at the level of orthologous genes, rather than focusing on species‐specific signatures of selection.

## 2 MATERIALS AND METHODS

### 2.1 Study species

*Arabis alpina* L. (Aal, alpine rock‐cress), *Arabidopsis halleri* (L.) Hayek (Aha, meadow rock‐cress), and *Cardamine resedifolia* L. (Cre, mignonette‐leaved bittercress) are all perennial, insect‐pollinated herbs in the Brassicaceae. *Arabis alpina* mostly grows in open and often calcareous habitats between 400 and 3,200 m a. s. l. It generally reproduces sexually, but exhibits substantial levels of inbreeding (Buehler et al., 2012; Tedder et al., 2011), and can propagate vegetatively via adventitious rooting (Mishra et al., 2020). Its genome size is 370–375 Mb with 2*n* = 16 chromosomes (Jiao et al., 2017; Willing et al., 2015). *Arabidopsis halleri* may clonally reproduce via stoloniferous growth, but in contrast to the two other species it is strictly outcrossing (Clauss & Koch, 2006). Its genome size is rather small (200–255 Mb; Briskine et al., 2017; Johnston et al., 2005) with 2*n* = 16. It can be found from 300 to about 2,300 m a. s. l. and is an extensively studied model system due to its tolerance to and hyperaccumulation of zinc and cadmium (e.g., Meyer et al., 2010; Sailer et al., 2018). Finally, *C. resedifolia* is a predominantly selfing species (Lihova et al., 2009) that grows mainly on siliceous substrates from 1,500 to above 3,000 m a.s.l. (Ometto et al., 2015). Reproductive isolation between the genus *Arabidopsis* and *Cardamine* was reported (Escobar‐Restrepo et al., 2007; Shimizu, 2002), suggesting that recent gene flow between the two genera as well as with further remotely related *Arabis* has not occurred.

### 2.2 Plant sampling and environmental data

For all three species, we sampled leaves of 20 plants from 18 populations (six populations per species) across the Alpine region in Switzerland (Figure 1b, Table S1) in summer 2010 and 2011 (total: 360 individuals). Leaves were immediately dried in silica gel. For studying convergent adaptation, one should ideally sample the species in the same habitats. However, this is often not possible in wild, nonexperimental set‐ups. Here, we thus concentrated on similar Alpine environmental gradients for each of the species. Our sampling design aimed to include three high/low altitude population pairs for each species. It has been shown that such a pairwise design has higher power to detect adaptation than a random or gradient sampling design even when analysed with an EAA approach that uses a continuous predictor, because it maximizes environmental differences while minimizing genetic differences due to gene flow between pairs (Lotterhos & Whitlock, 2015). Genomic data of all *A. halleri* populations were used in previous studies (Fischer et al., 2013, 2017; Rellstab et al., 2013, 2017). To minimize the risk of sampling the same genets (clonal individuals) multiple times, distance between sampled plants was at least 2 m. In addition to leaves, seeds were collected from some of the populations in order to grow plants for genome sequencing and assembly.

To describe the site conditions of the 18 populations, topoclimatic data were extracted from the high‐resolution (25 m) GIS layers of Zimmermann and Kienast (1999) using arcmap10 (ESRI). These climatic data, collected over a 30‐year period (1961–1990), are modelled and interpolated from a dense set of meteorological stations and high‐resolution topographic maps. Originally, 20 environmental factors were extracted and subsequently reduced to eight factors (Figure 1c, Table S1), which were not highly correlated among all 18 populations (Pearson's |*r*| < .7; Table S2a). These eight factors were aspect (ASPVAL), yearly precipitation (PRECYY), yearly frost days (SFROYY), slope (SLP25), yearly solar radiation (SRADYY), site water balance (SWB), yearly temperature (TAVEYY), and topographic wetness index (TWI25SS).

### 2.3 DNA extraction and genome sequencing

DNAs were extracted with the DNeasy Plant Kit (Qiagen, Hilden, Germany). DNA quality was assessed using 1.5% agarose gels stained with GelRed (Biotium, Fremont, USA) and a Nanodrop 8000 (Thermo Scientific, Waltham, MA, USA), and DNA quantity was determined with a Qubit fluorometer (dsDNA BR, Invitrogen, Carlsbad, CA, USA). We used population pools (7 μg RNA‐free genomic DNA in total) consisting of equimolar amounts of DNA from each of the 20 individuals sampled per population. These pools were also the basis for the reference genome assemblies of *A. alpina and C. resedifolia*. For each population pool, we prepared Illumina paired‐end libraries (2 × 100 bp) with an insertion size of ~250 bp. For the reference assembly of the highly outcrossing *A. halleri*, we made one Illumina paired‐end library from leaf material from a single individual grown from a seed of low‐polymorphism population Aha11 (for more details see Sailer et al., 2018). For the scaffolding, we further generated Illumina mate‐pair libraries (2 × 50 bp, insertion size 3,000 bp) from leaves of a single seed‐grown individual for each species (populations Aal19, Aha18, Cre14). Library preparation and sequencing were performed by GATC Biotech (Constance, Germany), the Quantitative Genomics Facility (D‐BSSE, ETH Zurich, Switzerland), and the Functional Genomics Center Zurich (University of Zurich and ETH Zurich, Switzerland) on the Illumina HiSeq 2000 platform (Illumina, San Diego, CA, USA).

### 2.4 Read processing and reference genome assemblies

cutadapt (Martin, 2011) was used to trim forward and reverse raw reads for tags and adapters. Phred‐type quality scores of Q20 were used for quality trimming with the fastx toolkit (http://hannonlab.cshl.edu/fastx_toolkit). Separately trimmed forward and reverse reads were then re‐synchronized to pairs with an in‐house Perl script (Fischer et al., 2013). Only paired sequences were used for further analysis.

For the de novo assemblies of *A. alpina* and *C. resedifolia*, quality trimmed paired‐end reads were assembled separately for each population pool with velvet 1.2.08 (Zerbino & Birney, 2008) using a range of *k*‐mer values. Assemblies were ranked based on N50 value, maximum scaffold length, total size of assembly, number of scaffolds and contigs, and BUSCO completeness values (Simao et al., 2015). The four best assemblies from this first step were scaffolded with their corresponding Illumina 3 kb mate‐pair libraries using SSPACE 2.3 (Boetzer et al., 2011), and gapfiller 1.9 (Boetzer & Pirovano, 2012) was used to close gaps. The final assessment and selection of the best assembly was based on the same parameters as above, the number of augustus (Stanke & Waack, 2003) predicted genes, and the number of reciprocal best blast hits compiled with BLAST + v2.2.23 (Camacho et al., 2009) and in‐house Perl scripts. Scaffolds below 200 bp in length were removed. The reference assembly of *A. halleri* (Ahalleri_CH_v2) was published in Sailer et al. (2018) and done in a slightly different way (see above); quality trimmed paired‐end and mate‐pair reads were assembled together with velvet 1.2.08.

### 2.5 Annotation

Automated gene prediction and structural annotations were generated with the automated pipeline MAKER2 (Holt & Yandell, 2011) using the gene prediction tools SNAP (Korf, 2004), augustus, and genemark‐es (Lomsadze et al., 2005). All proteins from the *A. thaliana* reference genome (TAIR10; Lamesch et al., 2012) were used as protein homology evidence. All expressed sequence tags (EST) for *A. thaliana* available on NCBI (https://www.ncbi.nlm.nih.gov/) were downloaded and included as alternative EST evidence. The model organism for repeat masking was set to *A. thaliana*. Two iterative MAKER2 runs were made to produce a final set of gene predictions and protein translations. Before starting the second iteration, a subset of the predicted genes from the first iteration was used to train and test the augustus gene prediction model.

For functional annotation, downloaded proteins from *A. thaliana* and the translated protein sequences resulting from the MAKER2 annotations of all three species were used as input for finding orthologous sequences with OMA (Altenhoff et al., 2015). Only orthologues with a one‐to‐one relationship with *A. thaliana* were selected; one‐to‐many and many‐to‐many relationships were discarded. The genes with one‐to‐one orthologues received the functional annotation of the corresponding orthologue in *A. thaliana*.

### 2.6 SNP calling

For the genome‐wide variant calling (SNPs, indels, and complex variants) of the 3x6 population pools, the sequencing reads were first mapped to the species‐specific de novo reference assembly with bwa aln 0.5.9 (Li & Durbin, 2009), allowing for a maximum edit distance of 10. picard tools 1.90 (http://broadinstitute.github.io/picard) were then used to add read‐group information, to mark PCR duplicates as well as to sort and to index the mapping files. The resulting BAM files were realigned with the "IndelRealigner" tool of GATK 2.5 (McKenna et al., 2010). A first round of variant calling was run with the "UnifiedGenotyper" tool of GATK. The same BAM files were also called with the "mpileup" tool of samtools (Li et al., 2009) and the output converted with the "mpileup2sync" and "snp‐frequency‐diff" tools of popoolation2 (Kofler et al., 2011). The variants from both GATK and samtools/popoolation2 were filtered with stringent parameters (minimum quality: 30; minimum counts of alternative allele across all populations: 6; minimum coverage: 20x; maximum coverage: 120x for *A. alpina*, 180x for *A. halleri*, and 160x for *C. resedifolia* to remove mappings to repeated sequences and to account for putatively paralogous regions). These high‐confidence variants were used for base quality recalibration with the GATK tool "BaseRecalibrator". A final variant calling round was then run with GATK "UnifiedGenotyper" on these recalibrated BAM files. The resulting variants were then filtered for biallelic SNPs, minimum quality of 30, minimum alternative allele frequency of 1%, and minimum coverage of 20x. The maximum coverage threshold was set as described above. Furthermore, we excluded all SNPs whose allele frequency estimates derived from SNP calling differed more than ±0.2 from the estimates calculated from read counts.

### 2.7 Population and landscape genomic analyses

We performed a principal component analysis (PCA) on allele frequencies of 210,000 randomly selected SNPs (see below) per species using the package factominer (Lê et al., 2008) in R 3.4.0 (R Development Core Team, 2018). To summarize genetic diversity, we calculated expected heterozygosity (*H*_e_) as in Fischer et al. (2017) using all SNPs. To quantify genetic differentiation, we used the R package poolfstat (Hivert et al., 2018) to calculate pairwise *F*_ST_ among populations of each species with the complete SNP sets. Pairwise *F*_ST_ matrices where then used to calculate a neighbour‐joining (NJ) tree with the "bionj" function of the R package ape (Popescu et al., 2012).

To identify signatures of selection, we applied two complementary approaches. First, outlier tests for directional selection targeted loci with large allele frequency differences among populations, assuming that locally beneficial alleles should have high frequencies in specific populations (Hohenlohe et al., 2010). Second, EAA aimed at identifying loci that are correlated to environmental predictors describing the habitat of populations (Rellstab et al., 2015). As a consequence of high SNP counts (up to 3.5 million per species; see Results) resulting in long computation times, we used high‐performance clusters (Brutus and Euler, ETH Zurich) to parallelize the analyses in 262, 251 and 195 random batches of around 14,000 SNPs for outlier tests, and 18, 17 and 14 random batches of around 210,000 SNPs for EAA in *A. alpina*, *A. halleri*, and *C. resedifolia*, respectively.

First, we used bayescan 2.1 (Fischer et al., 2011; Foll & Gaggiotti, 2008), an extension of the F‐model of Beaumont and Balding (2004), to detect *F*_ST_ outliers, i.e., loci that deviate from neutral expectations of locus‐specific population differentiation measures. bayescan estimates the posterior probability that each locus is under selection by testing two alternative models – one that includes the effect of selection and one that excludes it. Since bayescan cannot handle allele frequencies, we transformed the allele frequencies to the number of each allele at a given locus based on the number of chromosome sets included in the pools (*n* = 40 = sample size × 2 for diploid species). bayescan was run for each of the three species with standard parameters, i.e., 5,000 iterations, a thinning interval of 10, 20 initial pilot runs with a length of 5,000, and a burn‐in length of 50,000. Because we ran bayescan on up to 262 random subsets, we verified that all subruns converged to the same population specific *F*_ST_ value, and recalculated *q‐*values based on posterior probabilities using an in‐house script in R. We focused on outliers (false discovery rate [FDR] < 0.05) for directional selection, i.e. loci that exhibit positive *α*_i_‐values. We thus use the term "outlier" to describe outliers for directional selection only.

Second, we established latent factor mixed models (LFMM, Frichot et al., 2013) to test for linear associations between genetic variation (response variable) and environmental factors (explanatory variable), while controlling for neutral genetic structure with (random) latent factors. We used the command‐line version of LFMM 1.4 with population allele frequencies and 9,000 iterations after a burnin period of 1,000 iterations. Prior tests with one random batch of SNPs showed that run‐to‐run variation was very low (minimum Pearson's *r* = .999 for all pairwise *z‐*score comparisons; Figure S1) in all species. We therefore performed only one run per species for the final analysis. *p*‐Values were adjusted as described in François et al. (2016). Finally, we chose significant associations based on an FDR of 0.1% (*q* ≤ 0.001) using the R‐package qvalue (Storey & Tibshirani, 2003). For LFMM, the number of latent factors (*K*) has to be defined a priori. In our case, we chose to use *K* = 3 for all three species, based on the fact that we sampled three population pairs, and based on *p*‐value distribution across all environmental factors, which should be uniform to efficiently control for FDR (François et al., 2016). In sampling setups where neutral genetic and environmental variation are highly correlated, controlling for neutral structure can lead to the removal of adaptive signals in EAA (Yeaman et al., 2016). In each species, we therefore tested for a correlation of the first two principal components (PCs) of the PCA describing genetic structure (see above) with each of the eight environmental factors using Pearson correlations with the function "cor.test" in R.

Neither approach (bayescan and LFMM) accounts for differences in sequencing coverage among populations and SNPs, but since we applied stringent criteria for mapping, SNP calling, and filtering (which included stringent coverage thresholds) as described above, we assumed that our SNP allele frequencies were accurate. In natural populations of *A. halleri* (including populations Aha18 and Aha31 from this study), allele frequencies based on Pool‐Seq differed on average less than 4% from those based on individual genotyping (Rellstab et al., 2013).

### 2.8 Over‐representation of shared signatures of adaptation

For the bayescan analysis and EAA in each species, we considered a gene as a candidate for signature of adaptation if it could be functionally annotated and contained at least one SNP that was found to be an outlier for directional selection or associated with an environmental factor. The annotation criterion allowed us to identify candidate genes that were shared by all three species.

To test whether we find more shared signatures of adaptation among the three species than expected by chance (referred to as "over‐representation of shared signatures of adaptation" hereafter), we performed a resampling analysis to create a random empirical distribution of expected overlap and compared it to the observed overlap. In each of 10,000 iterations for *F*_ST_ outliers as well as for each environmental factor, we picked a random gene subset with a size equal to the species‐specific number of observed candidate genes from each species‐specific list of annotated genes. We then determined the number of shared genes among species. The resulting distribution of number of shared genes represents the random, empirical null distribution, and the proportion of observations above the real observed value denotes the empirical *p*‐value. In other words, empirical *p*‐values represent the probability of a value from a random draw to be above the observed value. If the *p*‐value was below .05, we considered this as a significant over‐representation of shared signatures of adaptation among the three species. We repeated this procedure for all pairs of species. This resampling approach was preferred to the alternative hypergeometric test (Yeaman et al., 2018), because it (i) can be applied to more than two species simultaneously; (ii) relies on assumption‐free and empirical distributions; and (iii) considers all species‐specific gene sets, and not only the common gene set, in comparisons. To compare among analyses, we also calculated the ratio of observed and (averaged) expected number of overlapping genes in those analyses that proved to be significant in the resampling approaches. It is important to note that this ratio does not represent an effect size of a statistical test (resampling analyses do not deliver effect sizes), but a relative quantification of over‐representation of shared signatures of adaptation.

### 2.9 Characterization of SNP variants and gene ontology analysis

We predicted the effects of all SNP variants using snpeff 4.3 (Cingolani et al., 2012), which classifies variants according to their predicted impact and effect (here nonsynonymous vs. synonymous) based on the annotation of the reference genome. We constructed the necessary snpeff databases for each species using GTF and FASTA files from our de novo reference assemblies. From the gene list with a shared signature of adaptation (present in all three species), we extracted a list of top candidate genes consisting of genes that have at least one nonsynonymous SNP that was associated to the same environmental factor or was a nonsynonymous bayescan outlier.

We tested for enrichment of nonsynonymous SNPs in genes with a shared signature of adaptation in all three species using a resampling analysis as described above. For each iteration (and for each environmental factor/outlier analysis), we randomly chose the number of genes with shared signatures of adaptation from the total shared gene set of all three species. We then determined how many of these randomly selected genes were also included in the total set of genes containing nonsynonymous SNPs in all three species. From this random, empirical null‐distribution, we again calculated the empirical *p*‐value. A *p*‐value below .05 indicates a significant enrichment of nonsynonymous SNPs in genes with signs of convergent adaptation.

Top candidate genes associated with environmental factors or having outlier SNPs were screened for hierarchical gene ontology (GO) over‐representation using the R package topgo (Alexa et al., 2006). Genes were annotated with locus identifier information from TAIR, and the total shared gene set of all three species was used as background reference list. Significance for each individual GO identifier was computed with Fisher's exact test and significant GO terms were identified at an FDR of 1%. Only GO terms having more than four and less than 1,000 associated genes were considered in analyses.

## 3 RESULTS

### 3.1 Next‐generation sequencing, reference assemblies, and SNP calling

Illumina sequencing generated 226,818,828 paired‐end reads and 124,015,090 mate‐pair reads for *A. alpina,* 76,269,120 paired‐end reads (population pools), 38,134,560 paired‐end reads (single individual, Aha_11_10B), and 113,073,812 mate‐pair reads for *A. halleri*, and 258,060,268 paired‐end reads and 284,061,676 mate‐pair reads for *C. resedifolia*. After quality trimming and filtering, 88% (*A. alpina*), 73% (*A. halleri*), and 80% (*C. resedifolia*) of the reads of the population pools (individual for *A. halleri*) were used for initial assemblies and mapping. The best assemblies, which are presented here, had a *k‐*mer value of 73 (*A. alpina*), 41 (*A. halleri*), and 67 (*C. resedifolia*). For the two species with known or estimated genome sizes, we were able to assemble de novo genomes accounting for 72% (*A. alpina*) and 82% (*A. halleri*) of the genome. In total, we could functionally annotate approximately 16,000 genes per species (Table 1), with an overlap of 12,485 genes among the three species. In the population pools, we identified 3,416,418 (*A. alpina*), 3,410,881 (*A. halleri*), and 2,733,931 (*C. resedifolia*) SNPs. Of these, 9.8% (*A. alpina*), 23.4% (*A. halleri*), and 8.6% (*C. resedifolia*) were located in annotated genes.

**Table 1:** Overview of the de novo assembled genomes of the three studied Brassicaceae species

	*Arabis alpina*	*Arabidopsis halleri* (Sailer et al., 2018)	*Cardamine resedifolia*
Assembly name	Aalpina_CH_v1	Ahalleri_CH_v2	Cresedifolia_CH_v1
Reference assembly, paired‐end	Aal05 (P)	Aha11 (I)	Cre20 (P)
Reference assembly, mate‐pair	Aal19 (I)	Aha18 (I)	Cre14 (I)
Assembly size	268.5 Mbp	164.6 Mbp	192.8 Mbp
Number of scaffolds	87,633	40,345	42,839
N50	25,182 bp	82,799 bp	48,548 bp
Largest scaffold	226.6 kb	774.2 kb	828.9 kb
Predicted genes larger than 67 amino acids	28,020	26,249	23,971
Predicted genes with functional annotation	15,909	16,088	16,047
BUCSO complete orthologues/proteins	1,377 (95.6%)/1,382 (96.0%)	1,327 (92.1%)/1,319 (91.6%)	1,402 (97.4%)/1,405 (97.6%)
BUSCO complete and single‐copy orthologues/proteins	1,341 (93.1%)/1,349 (93.7%)	1,311 (91.0%)/1,300 (90.3%)	1,381 (95.9%)/1,382 (96.0%)
BUSCO complete and duplicated orthologues/proteins	36 (2.5%)/33 (2.3%)	16 (1.1%)/19 (1.3%)	21 (1.5%)/23 (1.6%)
BUSCO fragmented orthologues/proteins	24 (1.7%)/29 (2.0%)	51 (3.5%)/73 (5.1%)	15 (1.0%)/16 (1.1%)
BUSCO missing orthologues/proteins	39 (2.7%)/29 (2.0%)	62 (4.4%)/48 (3.3%)	23 (1.6%)/19 (1.3%)

Abbreviations: I, individual sequencing data; P, Pool‐Seq data.

### 3.2 Population structure and genetic diversity

The PCAs describing genetic structure among populations showed that in *A. alpina* and (partly) in *C. resedifolia*, population pairs clustered together on PC1 (Figure S2). This was not the case in *A. halleri*, where altitudinal pairs were not that obvious. A similar pattern was evident in the NJ trees (Figure S3). Genetic diversity (*H*_e_; Table S3) was highest in the strictly outcrossing *A. halleri* (mean = 0.197 ± 0.015 *SD*), followed by *A. alpina* (0.138 ± 0.040) and *C. resedifolia* (0.120 ± 0.076), which showed the highest variation (*SD*) in genetic diversity among populations. Consequently, pairwise *F*_ST_ (Table S4) values were highest (and most variable) in the inbreeding *C. resedifolia* (mean = 0.481 ± 0.192), followed by *A. alpina* (0.317 ± 0.111) and *A. halleri* (0.081 ± 0.031).

Genetic structure, represented by PCAs (Figure S2), was not significantly correlated to environmental conditions in all three species (Table S5), with the exception of radiation and topographic wetness index for PC2 in *A. halleri*. Therefore, correcting for genetic structure should not reduce the adaptive signal in our analysis.

### 3.3 bayescan outlier analyses

We identified ~12,000 (*C. resedifolia*), ~43,000 (*A. alpina*), and ~61,000 (*A. halleri*) bayescan outlier SNPs for each species (Figure S4, Table S6). These outlier SNPs were positioned in 670 (*C. resedifolia*), 1,883 (*A. alpina*), and 3,863 (*A. halleri*) annotated genes (referred to as "outlier genes" hereafter; Table 2). Of these, 27 genes contained outlier SNPs in all three species (Table 2).

**Table 2:** Overlap among the three studied Brassicaceae species in the outlier and environmental association analyses (EAA). Number of genes containing bayescan outlier (top) and environment‐associated (below) SNPs in EAA (see also Figure S5) and overlap among the species. Aal, *Arabis alpina*; Aha, *Arabidopsis halleri*; Cre, *Cardamine resedifolia*. The last two columns list characteristics of genes shared in all three species (Aal∩Aha∩Cre): the number of genes with nonsynonymous (NS) SNPs and the number of genes with nonsynonymous SNPs that are associated to the same environmental factor or with nonsynonymous outlier SNPs are given

Analysis	Aal	Aha	Cre	Aal∩Aha	Aha∩Cre	Cre∩Aal	Aal∩Aha∩Cre	Aal∩Aha∩Cre	
								with NS SNPs	with EAA/outlier NS SNPs
bayescan outliers	1,883	3,864	670	422	155	80	27	21	1
ASPVAL	617	2,894	5,944	108	1,055	239	47	38	2
PRECYY	7,386	4,306	4,627	1,799	1,257	2,043	586	502	82
SFROYY	2,626	3,118	3,809	468	764	596	123	107	18
SLP25	6,853	3,053	5,313	1,206	988	2,189	462	399	50
SRADYY	2,954	4,077	5,842	681	1,506	1,049	280	247	21
SWB	5,434	6,196	4,370	1,932	1,602	1,409	565	504	81
TAVEYY	5,398	4,302	2,919	1,380	826	930	283	247	34
TWI25SS	4,931	5,577	5,266	1,569	1,766	1,552	581	488	90
Unique in all three species							2,050	1,759	298

Abbreviations: ASPVAL, aspect; PRECYY, yearly precipitation sum; SFROYY, annual average of frost days; SLP25, slope; SRADYY, yearly solar radiation; SWB, site water balance; TAVEYY, yearly temperature; TWI25SS, topographic wetness index.

For details on environmental factors see Table S1.

### 3.4 Environmental association analyses

In LFMM, 4.40% (*A. alpina*), 1.64% (*A. halleri*), and 4.55% (*C. resedifolia*) of the up to 27.3 million tested associations per species were significant (Table S7). Associations were found in 17.31% (*A. alpina*), 7.29% (*A. halleri*), and 15.78% (*C. resedifolia*) of all SNPs. Overall, 83,635 (*A. alpina*), 161,816 (*A. halleri*), and 399,680 (*C. resedifolia*) SNPs in functionally annotated genes were associated to one of the eight environmental factors (Table S8), resulting in 11,447 (*A. alpina*), 10,205 (*A. halleri*), and 10,615 (*C. resedifolia*) annotated genes that contained at least one associated SNP. SNPs from annotated genes had proportionally and consistently more associations than those that were not annotated (Table S7 and S8), but the difference was not significant (paired *t‐*test, *t *= –2.477, *p* =  .13). Each species differed in the proportion of SNPs that was associated to environmental factors (Table 2). For *A. halleri*, precipitation and slope had the highest numbers of associated genes (Figure S5). Associations for *A. alpina* were dominated by site water balance and topographic wetness index, and those of *C. resedifolia* by aspect and radiation (Figure S5). Overlap among species was considerable; between 108 and 2,189 genes were present in associations with the same factor in species pairs (Table 2). Most importantly, we found between 47 (aspect) and 586 (precipitation) genes that were associated to the same environmental factor in all three species (Table 2).

### 3.5 Shared signatures of adaptation

**FIGURE 2:**
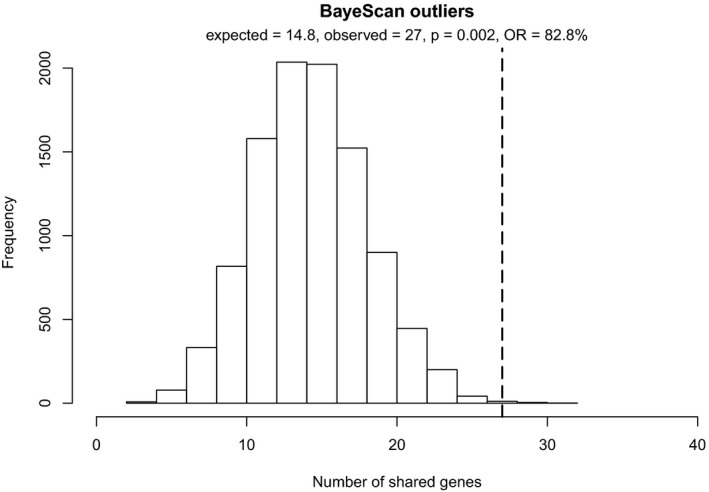
Shared signatures of adaptation in bayescan outlier genes. Shown is the number of shared outlier genes among *Arabis alpina*, *Arabidopsis halleri*, and *Cardamine resedifolia* (dashed line, 27 genes) compared to random subsamples using the complete gene lists, iterated 10,000 times. Given is the average expected number of shared genes based on random subsampling, the observed number of shared genes, the empirical *p*‐value, and the relative over‐representation (OR) of shared signatures as compared to the expected value

**FIGURE 3:**
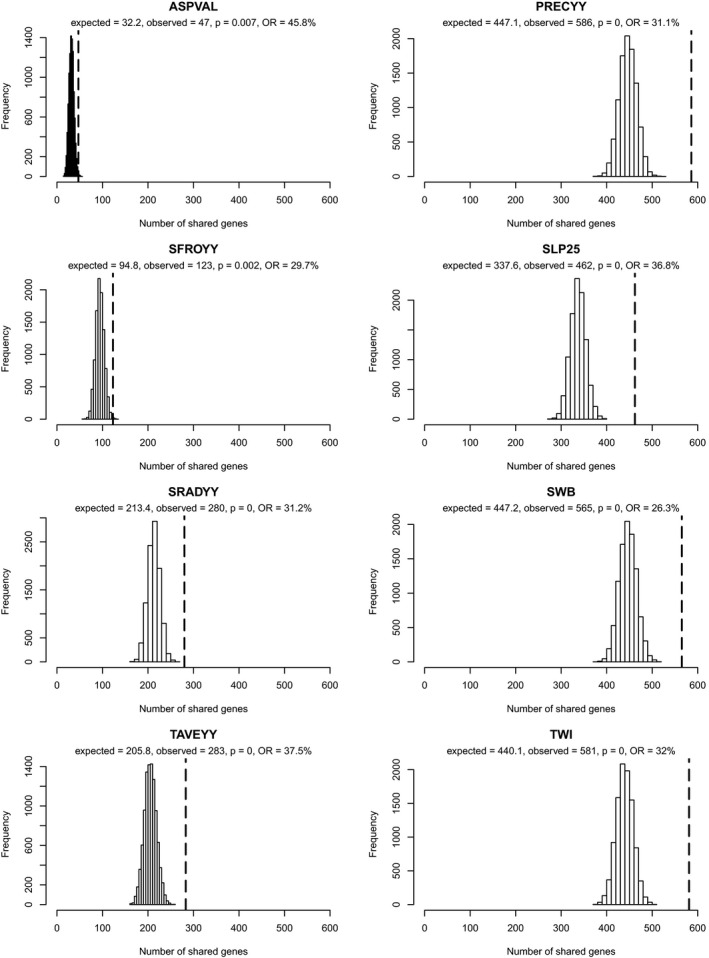
Shared signatures of adaptation in genes associated with environmental factors. For each environmental factor, the number of shared associated genes (dashed line) compared to 10,000 iterated random subsamples using the complete gene lists is shown. Given is the average expected number of shared genes based on random subsampling, the observed number of shared genes, the empirical *p*‐value, and the relative over‐representation (OR) of shared signatures as compared to the expected value. ASPVAL, aspect; PRECYY, yearly precipitation; SFROYY, yearly frost days; SLP25, slope; SRADYY, yearly solar radiation; SWB, site water balance; TAVEYY, yearly temperature; TWI25SS, topographic wetness index. For more details on environmental factors see Table S1

Genes that contained bayescan outlier SNPs or SNPs associated to the same environmental factor in all three species were significantly (resampling analysis, empirical *p* < .05) over‐represented compared to random expectations (Figures 2 and 3). Shared outlier genes were 83% more frequent than expected by chance alone. In only 16 out of 10,000 random subsamples (empirical *p* = .002), the number of shared genes was higher or equal to the observed number of 27 shared outlier genes (Figure 2). The same significant over‐representation was found for genes that were associated to environmental factors (Figure 3), although relative over‐representation was lower than for outlier genes (between 26.3% and 45.8% depending on the environmental factor). The highest relative over‐representation was found in aspect (45.8%), but it was lowest in terms of the number of absolute observed shared genes (47) compared to the expected number (32.2; Figure 3). In this respect, topographic wetness index (581 vs. 440.1) and precipitation (586 vs. 447.1) had the highest over‐representation.

Likewise, in all pairwise comparisons, the over‐representation of shared adaptation signatures was significant for outlier and associated genes (empirical *p* <  .05), except for aspect in the comparison of *A. alpina* and *A. halleri* (empirical *p* = .057; Table 3). The highest relative over‐representation of shared signatures was consistently found for *A. halleri* and *C. resedifolia*, the species pair with the shortest phylogenetic distance, except for outlier genes and for genes associated with aspect. For the outlier and aspect‐associated genes, the comparison between *A. alpina* and *C. resedifolia* had the highest relative over‐representation of shared adaptation signatures. The lowest relative over‐representation was typically found in comparisons of *A. alpina* and *A. halleri*.

**Table 3:** Shared signatures of adaptation in pairs of the three studied Brassicaceae species. Numbers derive from bayescan outlier (top) and environmental association (bottom) analyses. Given is the average expected number of shared genes based on random subsampling, the observed number of shared genes, and the relative over‐representation (OR in %) of the shared signature compared to the expected value

Analysis	Aal vs. Aha			Aha vs. Cre			Aal vs. Cre		
	Expected	Observed	OR	Expected	Observed	OR	Expected	Observed	OR
bayescan outliers	381.6	422	10.5	139.0	155	11.5	68.8	80	16.4
ASPVAL	93.5	108^1^	—	922.1	1,055	14.4	199.6	239	19.7
PRECYY	1666.7	1,799	7.9	1069.2	1,257	17.6	1860.9	2,043	9.8
SFROYY	429.5	468	9.0	637.1	764	19.9	543.5	596	9.7
SLP25	1097.8	1,206	9.9	870.9	988	13.4	1981.9	2,189	10.4
SRADYY	630.7	681	8.0	1277.7	1,506	17.9	939.9	1,049	11.6
SWB	1766.3	1,932	9.4	1452.4	1,602	10.3	1293.2	1,409	9.0
TAVEYY	1218.6	1,380	13.2	673.8	826	22.6	858.7	930	8.3
TWI25SS	1440.6	1,569	8.9	1577.0	1,766	12.0	1413.8	1,552	9.8

Note

All observed values are significantly (empirical *p* < .05) higher than the expected values, except for ASPVAL in the comparison^1^ of Aal and Aha.

For details on environmental factors see Table S1

Abbreviations: Aal, *Arabis alpina*; Aha, *Arabidopsis halleri*; ASPVAL, aspect; Cre, *Cardamine resedifolia*; PRECYY, yearly precipitation sum; SFROYY, annual average of frost days; SLP25, slope; SRADYY, yearly solar radiation; SWB, site water balance; TAVEYY, yearly temperature; TWI25SS, topographic wetness index.

### 3.6 Characterization of SNP variants

snpeff predicted the effect of SNPs in 12,485 shared genes in all three species. Of those, 8,681 genes (69.5%) contained nonsynonymous SNPs in all three species. This proportion of nonsynonymous SNPs was higher in candidate genes: 77.8% of the shared bayescan outlier genes and an average of 86.1% of the shared EAA genes contained nonsynonymous SNPs (Table 2). This enrichment of nonsynonymous SNPs in candidate genes was statistically significant for all EAA factors (empirical *p* < .05; Table S9), but not for outlier genes (empirical *p *= .12). In total, we identified one bayescan outlier gene that contained nonsynonymous SNPs in all three species and 297 genes with nonsynonymous SNPs associated to the same environmental factor in all three species. We considered these 298 genes as top candidate genes for convergent adaptation to the Alpine environment (Table S10). The GO term enrichment analysis using the 297 environmentally‐associated top candidates identified no significant GO terms at an FDR of 1%. However, some GO terms were close to the significance threshold. The seven most strongly enriched GO terms (FDR < 2%) were (in this order): "enzyme linked receptor protein signaling pathway", "transmembrane receptor protein tyrosine kinase signaling pathway", "cell surface receptor signaling pathway", "phosphorylation", "RNA modification", "protein phosphorylation", and "defence response signaling pathway".

## 4 DISCUSSION

Empirical studies using genetic crosses and sequencing approaches have shown that related lineages and species may display some degree of convergent genetic and phenotypic adaptation to similar environmental conditions and gradients (e.g., Colosimo et al., 2005; Conte et al., 2012; Prunier et al., 2012). Studies screening the whole genome or exome of different species, however, are still largely restricted to model species (e.g., Zhao & Begun, 2017), with some exceptions (e.g. Guggisberg et al., 2018; Yeaman et al., 2016). Here, we used two types of genome scans (*F*_ST_ outlier tests and EAA) in three Brassicaceae species to test whether the same genomic regions were involved in adaptation to heterogeneous environmental conditions in the Alps. As main outcomes of our study, we found a large number of species‐specific genomic signatures of selection, but at the same time a significantly higher amount of shared adaptation signatures than expected by chance. The highest relative over‐representation of shared adaptation signatures was discovered in the two most closely related species. Moreover, we detected a higher proportion of nonsynonymous SNPs in identified candidate genes for convergent adaptation than across the whole genomes.

We based the genomic analyses on our own de novo assembled reference genomes (Table 1 and Sailer et al., 2018) despite two of the three species (*A. alpina* and *A. halleri*) having published reference genomes (Briskine et al., 2017; Jiao et al., 2017). This approach ensured inference compatibility among species by using similar sequencing strategies and analytical pipelines, leading to the same potential biases in all three data sets. Although our reference genomes are more fragmented than the published ones, they were based on local accessions (the accessions for the published reference genomes of *A. alpina* and *A. halleri* derived from Spain and Japan, respectively), keeping mapping errors and biases at a minimum. Our reference genomes showed a high proportion of completeness (Table 1) and contained up to 28,020 predicted genes, of which ~16,000 could be functionally annotated. Our analyses strongly profited from the wealth of resources available for the related model species *A. thaliana* (Kaul et al., 2000; Lamesch et al., 2012). For unbiased comparability, we concentrated inferences on genes that could be functionally annotated to these resources.

In the single‐species genome scans, up to 1.8% and 17.3% of all SNPs in the *F*_ST_ outlier tests and EAAs (all environmental factors combined), respectively, showed a signature of selection, leading to a high number (but see Ahrens et al., 2018) of putatively adaptive genes (Table 2). On the one hand, some of these genes might represent false positives, e.g., due to not accounting for linkage disequilibrium, violations of assumptions of the genome scan methods (Lotterhos & Whitlock, 2014, 2015; de Villemereuil et al., 2014), and the fact that we considered a gene as a candidate when it contained at least one outlier or associated SNP. On the other hand, complex and highly polygenic patterns are expected in climate adaptation (Csillery et al., 2018; Yeaman et al., 2016). In any case, we accounted for confounding population structure, stringently controlled for false discoveries due to multiple testing, and, most importantly, concentrated on shared signatures of adaptation of three species rather than single‐species results. The latter should substantially reduce false positive findings, because convergent signatures as a result of processes other than selection (e.g., mutations, drift) are highly unlikely (Conte et al., 2012; Losos, 2011; Yeaman et al., 2016). Drift is a random, stochastic process and should therefore differ among distinct species. Finally, false positive genes from single‐species analyses should rather lead to an underestimation of over‐representation of shared signatures of adaptation (Yeaman et al., 2018).

We found that shared signatures of adaptation in all species were significantly over‐represented, with 26.3% (genes associated with site water balance) to 82.8% (outlier genes) more genes with shared adaptation signatures than expected by chance. Among environmental factors, these proportions did not differ substantially (26.3%–45.8%). However, environmental factors related to water availability (precipitation, site water balance, topographic wetness index) had the highest number of shared adaptation genes, mainly because they exhibited the highest numbers in single‐species analyses. This finding strengthens earlier studies in these three species that identified precipitation‐related environmental factors as key drivers of local adaptation in Alpine environments (e.g., Fischer et al., 2013; Poncet et al., 2010; Rellstab et al., 2017). However, many studies looking at the genomics basis or phenotypic characteristics of local adaptation in the study species concentrated on altitudinal (e.g., Kubota et al., 2015; Lobreaux & Miquel, 2020; Ometto et al., 2015; de Villemereuil et al., 2018; Wingler et al., 2015) and latitudinal gradients (e.g., Toräng et al., 2015), therefore mainly targeting temperature, frost, and radiation differences. In the present study, these factors did not exhibit an exceptionally high number of associated genes. This finding indicates the strong influence of sampling design on the outcome of adaptation studies, emphasizing the use of similar environmental ranges for comparative studies.

How much evidence for convergent adaptive evolution can be expected among species? On the one hand, convergence is expected in closely related species as a consequence of similar demographic histories, a similar pool of standing genetic variation, similar genetic mechanisms that influence a phenotype, and a similar genetic background (Conte et al., 2012). Conte et al. (2012) analysed populations that diverged between 100 years and one million years ago, and species that separated between 100,000 years and 100 million years ago. These authors found higher probabilities of repeated gene use in convergent phenotypic evolution in more closely related compared to more distantly related lineages/species, and higher probabilities to detect these patterns in candidate gene approaches compared to studies based on quantitative trait locus (QTL) analyses in genetic crosses. Whole‐genome sequencing approaches like the one used in the present study might have convergence levels closer to QTL analyses than to candidate gene approaches, because the former approaches cover a much broader genomic space than when testing only a limited number of candidate genes. Convergence can also be expected in similar environments, because they exert similar selection pressures, generating nonrandom and repeated outcomes in independent lineages (Stuart, 2019). On the other hand, convergent adaptation is not necessarily expected in heterogeneous Alpine environments, because heterogeneity exerts complex multifactorial selection pressures that might be geographically restricted (Rellstab et al., 2017). In our case, environmental ranges were not identical, but considerably overlapping among the three species (Figure 1c). Moreover, polygenic adaptation is expected to contribute substantially to adaptation in heterogeneous environments, thus reducing the probability of identifying convergence signatures as a result of genetic constraints (Yeaman et al., 2018). Most of the prominent examples of convergence in the literature investigated monogenic convergence (e.g., Colosimo et al., 2005; Jones et al., 2012), but (presumably) polygenic convergence among species has also been reported (e.g., Yeaman et al., 2016).

The probability of convergence should decrease with increasing phylogenetic distance, because more distantly related species use different developmental pathways and gene networks to adapt to new or changing environments (Conte et al., 2012). Moreover, pleiotropic constraints and the supply of beneficial mutations at a locus probably depend on its genetic background. Both had more time to diverge in species that separated a long time ago. Indeed, a meta‐analysis showed that the probability of convergence increases with decreasing divergence time between lineages (Conte et al., 2012). This was also confirmed in our study. The most closely related species pair, *A. halleri* and *C. resedifolia*, showed the highest relative over‐representation of shared adaptation signatures in pairwise comparisons, although *A. halleri* and *A. alpina* were sampled in more similar environments than *C. resedifolia* (Figure 1c).

Our list of top candidate genes involved in convergent adaptation (Table S10) contains 298 genes with nonsynonymous SNPs, which were bayescan outliers or associated with the same environmental factors in all three species. At first sight, this list of shared genes looks arbitrary; it mostly lacks typical genes involved in the response to abiotic stress and comprises no significantly enriched GO terms at an FDR of 1%. One possible explanation for this finding is that we concentrated on genes that could be annotated in the TAIR database. Therefore, we might have missed species‐specific, nonannotated genes. Even though we believe this to be rather unlikely, perhaps it was exactly these genes that were involved in response to abiotic stress. Another possibility is that shared adaptation rather builds on basic processes (transcription regulation, metabolic and catabolic processes, etc.), and that typical abiotic stress reactions are species‐specific. Finally, information found in GO databases like TAIR are generally derived from a cellular perspective and ignore ecologically derived gene annotation (Landry & Aubin‐Horth, 2007; Pavy et al., 2017; Rellstab et al., 2015). Still, the fact that genes share adaptation signatures in all three species makes it very unlikely that they represent false positives, and the enrichment of nonsynonymous mutations (indicating functional relevance) strongly underlines their role in adaptation to abiotic factors.

Nevertheless, there were several interesting genes identified as top candidates that are known to be related to abiotic conditions. For example, *SIZ1* (AT5G60410), encoding a DNA‐binding protein with MIZ/SP‐RING zinc finger, contained at least one nonsynonymous SNP in all three species and was associated to precipitation. *SIZ1* regulates abscisic acid signaling in the drought‐response pathway of *Arabidopsis* (Catala et al., 2007; Miura et al., 2009) as well as in drought response and freezing tolerance in several other plant species (e.g., in rice, Mishra et al., 2017). Another example is *UVH3* (AT3G28030), which is encoding a DNA repair enzyme for damage by UV irradiation (Liu et al., 2001). UV irradiation has long been discussed as a major environmental stress in altitudinal adaptation. The gene however, was associated to frost days in our analyses. Finally, nine of the 298 genes (AT1G05700, AT1G06000, AT1G06970, AT1G68720, AT1G80680, AT2G20960, AT2G30290, AT2G41890, AT3G19230; for names see Table S10) were identified as top candidates for topo‐climatic adaptation in *A. halleri* in a previous study (Fischer et al., 2013), notably using some of the populations described here. Three of these nine genes (AT1G05700, AT1G06970, AT1G80680) were confirmed of being associated to the same environmental factor in a much larger and independent population set of *A. halleri* (Rellstab et al., 2017). Of these genes, *SAR3* (AT1G80680) was even associated to the same environmental factor (site water balance) in all three studies (this study; Fischer et al., 2013; Rellstab et al., 2017). *SAR3* belongs to a gene family that plays a role in the plant hormone auxin pathway by suppression of the transcriptional repressors of the *AXR* gene family (Parry et al., 2006), which have a regulatory role for various proteins under drought stress in *Arabidopsis* (Bianchi et al., 2002). Given all this evidence, *SAR3* is a prime candidate for plant adaptation to water availability in Alpine environments.

One obvious question to ask is whether the shared patterns of adaptation stem from independent mutations, standing genetic variation, or hybridization (Stern, 2013). This question could be addressed by inspecting genomic regions surrounding the identified SNPs. For example, Lee and Coop (2017) developed a method that compares within‐ and between‐population co‐ancestry coefficients around selective peaks. However, such approaches rely on accurate among‐species alignments of sequences, which proofed to be difficult in our case (results not shown). On the one hand, our finding that the most closely related species pair exhibited the highest relative over‐representation of shared adaptation signatures supported the notion that in more closely related species, the same genes are more frequently involved in adaptive processes, presumably because adaptive evolution in closely related species is affected by the same constraints (Conte et al., 2012). On the other hand, the fact that most relevant SNPs were different in the three species suggests that adaptation builds on independent mutations in these genes.

Our study has some technical and analytical limitations, due in part to the fact that we compared different species. First, only shared genic regions that are annotated in a related model species were used. This approach ignores regions of the genome that, e.g., regulate gene expression or are species‐specific. Nevertheless, the use of shared genic regions allows a balanced comparison among the different species and lifts the analysis to the functional level of genomes. Second, due to the complexity of comparisons among three species, we concentrated on genes that represented one‐to‐one orthologues in the annotation. This approach ignores potential gene duplications, which might play a substantial role in adaptation processes (Seppey et al., 2019; Yeaman et al., 2016). Third, the three study species differ substantially in respect to mating system and genetic structure. Although both bayescan and LFMM account for confounding genetic structure, one cannot completely exclude that the differences in species' demography and life‐history traits were affecting the results. For example, for a species that generally shows high genetic differentiation among populations and low population‐specific genetic diversity, bayescan has less power to differentiate between drift and selection effects, and consequently to identify outlier SNPs under directional selection than for a species with only moderate population differentiation (Foll & Gaggiotti, 2008). This could have been the case in the mainly selfing *C. resedifolia* and, to a lesser extent, in the mixed‐mating *A. alpina*, where among‐population differentiation was higher (Table S4) than in *A. halleri*, leading to fewer outlier genes (Table 2).

In conclusion, besides large amounts of species‐specific signatures of adaptation, we found compelling evidence for convergent genetic adaptation to similar Alpine environmental gradients in three Brassicaceae species that diverged 13–23 million years ago. Studies showing such genetic convergence in whole genomes of natural populations of non‐model species have been rare so far. Therefore, our work helps to assess the level of convergent adaptation and to identify genes that show similar signatures of adaptation to the highly heterogenous Alpine environment.

## AUTHOR CONTRIBUTIONS

A.W., K.K.S., and R.H. acquired funding. M.C.F., C.R., A.W., F.G., K.K.S., and R.H. designed the study. F.G., C.R., and M.C.F. organized and performed sampling. M.C.F., C.R., C.S., and S.Z. conducted bioinformatic analyses. M.C.F., C.R., C.S., A.T., and S.Z. performed data analysis. C.R. wrote the manuscript, with input from all coauthors. All authors approved the final version of this manuscript.

## Supporting information

Figures S1–S5
Tables S1–S10

## Data Availability

The reference genome assemblies (and their corresponding raw reads) can be found at NCBI (www.ncbi.nlm.nih.gov) under PRJNA534217 (*A. alpina*), PRJNA492199 (*A. halleri*), and PRJNA541873 (*C. resedifolia*). Raw reads of population pools can be found at European Nucleotide Archive (ENA, www.ebi.ac.uk/ena) under PRJEB38197 (*A. alpina*) and PRJEB38200 (*C. resedifolia*). For *A. halleri*, raw reads for population pools can be found under PRJEB18647 (ENA) and SRP029378 (NCBI). Functional and structural annotation of the reference assemblies, and population allele frequencies and read counts are available at the Dryad digital repository: https://doi.org/10.5061/dryad.4mw6m9081.
