# Supplementary material for: Genomic signatures of convergent adaptation to Alpine environments in three Brassicaceae species
Source: Mol Ecol. 2020 Oct 14;29(22):4350–65. doi: 10.1111/mec.15648 (PMC7756229; doi:10.1111/mec.15648)
Supplement: Supplementary file 1 — Figures S1–S5 Tables S1–S10 [file MEC-29-4350-s001.docx]

**Supplemental Information for:**

**Genomic signatures of convergent adaptation to Alpine environments in three Brassicaceae species**

Christian Rellstab, Stefan Zoller, Christian Sailer, Andrew Tedder, Felix Gugerli, Kentaro K. Shimizu,
Rolf Holderegger, Alex Widmer & Martin C. Fischer

**Table of Contents:**

| **Figure S1: Run-to-run variation in LFMM analyses.** | Page 2 |
| --- | --- |
| **Figure S2: Neutral population structure of the three studied species.** | Page 3 |
| **Figure S3: Neighbor-joining trees illustrating genetic distances among populations of the three studied species.** | Page 4 |
| **Figure S4: Results of BayeScan outlier analyses.** | Page 5 |
| **Figure S5: Importance of environmental factors inferred by the environmental association analyses for each species.** | Page 6 |
| **Table S1: Environmental characteristics of the 18 populations used in the study.** | Page 7 |
| **Table S2: Pairwise correlation matrix of the eight environmental factors used in environmental association analyses.** | Page 8 |
| **Table S3: Genetic diversity in the 18 study populations.** | Page 9 |
| **Table S4: Genetic differentiation among the 6 study populations per species.** | Page 10 |
| **Table S5: Comparison of population structure and environmental conditions.** | Page 11 |
| **Table S6: Overview of BayeScan outlier analyses.** | Page 12 |
| **Table S7: Results of environmental association analyses for all SNPs.** | Page 13 |
| **Table S8: Results of environmental association analyses for annotated SNPs.** | Page 14 |
| **Table S9: Observed and expected number of shared genes with non-synonymous SNPs.** | Page 15 |
| **Table S10: List of the 298 top candidate genes.** | Page 16 |

**Figure S1: Run-to-run variation in LFMM analyses.** For each species, one random batch of 210,000 SNPs was used in three independent runs and the resulting *z*-scores compared. Aal *= Arabis alpina*; Aha *= Arabidopsis halleri*; Cre *= Cardamine resedifolia.*


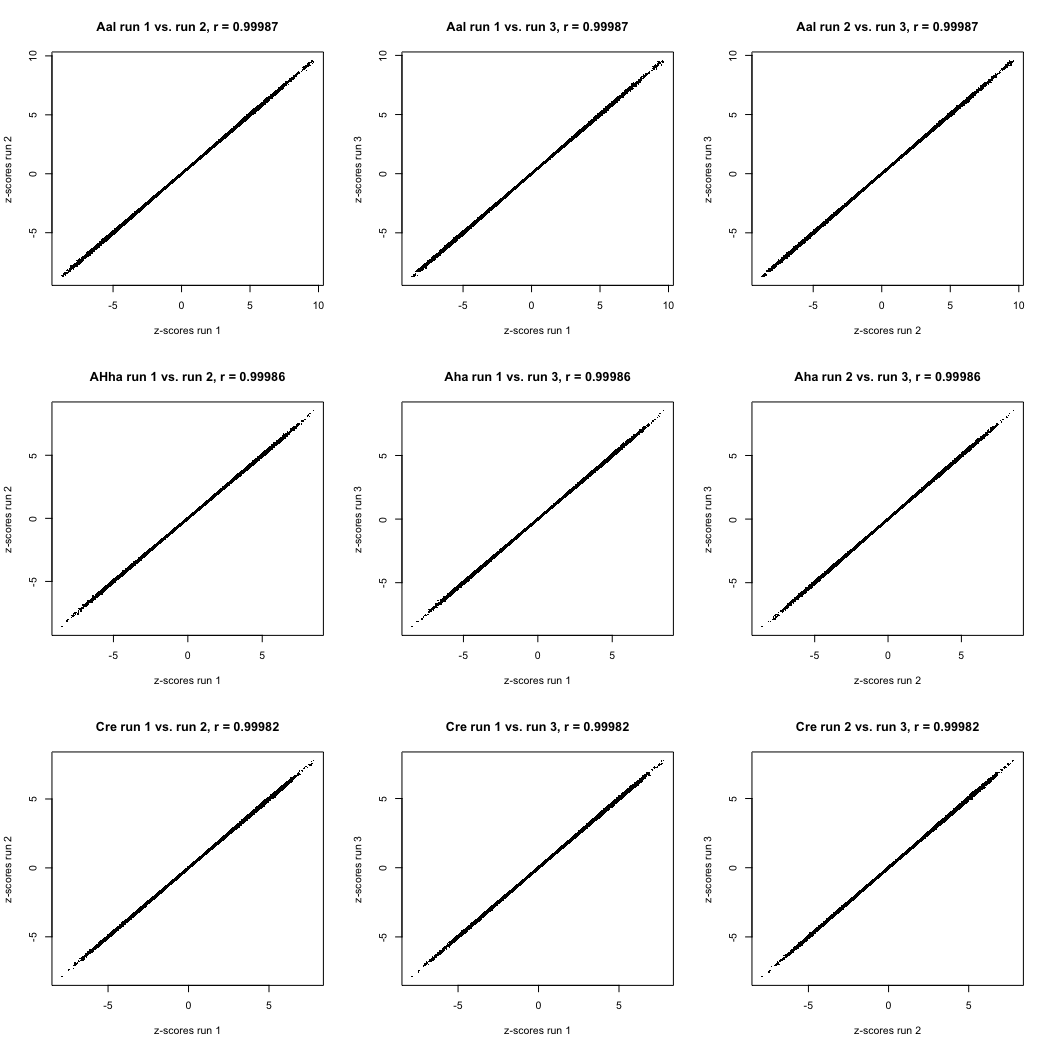


**Figure S2: Neutral population structure of the three studied species.** Shown are the two first axes of a principal component analysis (PCA) using population allele frequencies of 210'000 randomly selected SNPs.

**Figure S3: Neighbor-joining trees illustrating genetic distances among populations of the three studied species.** Based on pairwise *F*_ST_ values (Table S4) using all SNPs.

**Figure S4: Results of BayeScan outlier analyses.** Shown are log-scaled BayeScan posterior odds (PO, in green) and *q* values (in blue) of 2.7 to 3.4 million *F*_ST_ values of the SNPs of the three species. The histograms in the lower part show the distribution of PO and *q* values for each species. The vertical red line in the *q* value plot represents the FDR threshold of 5% used to identify outlier SNPs. Only significant SNPs under directional selection (positive ⍺_i_ value) were used for further analyses.

**Figure S5: Importance of environmental factors inferred by the environmental association analyses for each species.** Given is the proportion of genes associated to the specific environmental factor in respect to the factor with the highest number of associated genes in that species. ASPVAL = aspect; PRECYY = yearly precipitation sum; SFROYY = annual average of frost days; SLP25 = Slope; SRADYY = yearly solar radiation; SWB = site water balance; TAVEYY = yearly temperature; TWI25SS = topographic wetness index. For details on environmental factors see Table S1.

**Table S1: Environmental characteristics of the 18 populations used in the study.** Given are plant species, population code, coordinates, elevation (digital elevation model DHM25 L2), and values of the eight environmental factors used in environmental association analyses. ASPVAL = aspect [°]; PRECYY = yearly precipitation sum [1/10 mm/year]; SFROYY = annual average of frost days [d]; SLP25 = Slope [°]; SRADYY = yearly solar radiation [kJ/m^2^/day]; SWB = site water balance [1/10 mm/year]; TAVEYY = yearly temperature [1/100 °C]; TWI25SS = topographic wetness index (potential soil humidity [-]). A single individual of population Aal19* and Aha18** was used for the assembly of the reference genome of *Arabis alpina* and *Arabidopsis halleri*, respectively (mate-pair sequencing).

| **Species** | **Population** | **Latitude [°N]** | **Longitude [°E]** | **Elevation [m a.s.l.]** | | **ASPVAL** | **PRECYY** | **SFROYY** | **SLP25** | **SRADYY** | **SWB** | **TAVEYY** | **TWI25SS** |
| --- | --- | --- | --- | --- | --- | --- | --- | --- | --- | --- | --- | --- | --- |
| *Arabis alpina* | Aal05 | 47.09909 | 9.05408 | 554 |  | 47 | 17,348 | 0 | 28 | 18,326 | 240 | 801 | -121 |
|  | Aal18 | 47.16862 | 9.14544 | 1,534 |  | 99 | 23,204 | 23 | 10 | 19,246 | 230 | 317 | -161 |
|  | Aal27 | 46.97581 | 9.40496 | 2,295 |  | 54 | 17,690 | 120 | 14 | 18,439 | 400 | -43 | 31 |
|  | Aal31 | 47.04009 | 9.39734 | 712 |  | 71 | 13,262 | 3 | 41 | 17,699 | 29 | 738 | -151 |
|  | Aal34 | 47.06786 | 9.02061 | 1,954 |  | 99 | 21,982 | 43 | 14 | 17,119 | 0 | 103 | -85 |
|  | Aal36 | 47.15436 | 9.30674 | 2,300 |  | 24 | 21,381 | 170 | 6 | 18,321 | 0 | -61 | -214 |
|  | Aal19* | 47.03512 | 8.99197 | 861 |  |  |  |  |  |  |  |  |  |
| *Arabidopsis halleri* | Aha09 | 46.36925 | 9.65868 | 1,406 |  | 83 | 11,368 | 25 | 6 | 16,865 | -44 | 527 | -57 |
|  | Aha11 | 46.27767 | 10.10618 | 1,070 |  | 81 | 10,561 | 40 | 15 | 16,654 | -1,118 | 810 | 49 |
|  | Aha19 | 46.41125 | 10.02253 | 2,307 |  | 90 | 15,564 | 74 | 5 | 19,180 | 380 | 60 | -141 |
|  | Aha21 | 46.36682 | 9.63081 | 1,611 |  | 65 | 12,202 | 31 | 21 | 19,780 | 231 | 431 | -173 |
|  | Aha31 | 46.33682 | 9.52171 | 798 |  | 88 | 13,133 | 10 | 6 | 18,000 | -163 | 892 | -3 |
|  | AhaN3 | 46.49926 | 9.82731 | 2,067 |  | 57 | 13,008 | 108 | 25 | 20,201 | 400 | 74 | -144 |
|  | Aha18** | 46.41593 | 8.82605 | 865 |  |  |  |  |  |  |  |  |  |
| *Cardamine resedifolia* | Cre03 | 46.57955 | 9.81853 | 2,201 |  | 6 | 12,478 | 159 | 6 | 15,560 | 0 | 4 | 108 |
|  | Cre12 | 46.48544 | 9.77279 | 2,863 |  | 6 | 12,890 | 594 | 30 | 12,955 | 0 | -320 | -356 |
|  | Cre13 | 46.55621 | 8.82969 | 2,422 |  | 100 | 15,282 | 205 | 30 | 21,961 | 380 | -50 | -178 |
|  | Cre14 | 46.39060 | 9.10911 | 1,826 |  | 52 | 17,287 | 0 | 18 | 19,240 | 365 | 334 | -16 |
|  | Cre18 | 45.98368 | 7.78689 | 3,111 |  | 3 | 15,601 | 891 | 5 | 17,119 | 0 | -317 | -341 |
|  | Cre20 | 46.18153 | 7.69320 | 2,004 |  | 14 | 10,269 | 36 | 27 | 13,022 | -210 | 228 | -26 |

**Table S2: Pairwise correlation matrix of the eight environmental factors used in environmental association analyses.** Given is the Pearson correlation coefficient *r*. (a) Three species combined; (b-d) for each species separately. For details on environmental factors, see Table S1.

| (a) Three species combined   \|  \| PRECYY \| SFROYY \| SLP25 \| SRADYY \| SWB \| TAVEYY \| TWI25SS \| \| --- \| --- \| --- \| --- \| --- \| --- \| --- \| --- \| \| ASPVAL \| 00.23 \| −0.58 \| −0.03 \| 0.60 \| 0.05 \| 0.47 \| 0.21 \| \| PRECYY \|  \| −0.03 \| −0.25 \| 0.36 \| 0.40 \| −0.24 \| −0.21 \| \| SFROYY \|  \|  \| −0.11 \| -0.28 \| 0.00 \| −0.69 \| −0.67 \| \| SLP25 \|  \|  \|  \| -0.03 \| 0.12 \| 0.15 \| −0.22 \| \| SRADYY \|  \|  \|  \|  \| 0.51 \| 0.11 \| −0.04 \| \| SWB \|  \|  \|  \|  \|  \| −0.38 \| −0.27 \| \| TAVEYY \|  \|  \|  \|  \|  \|  \| 0.47 \| | (b) *Arabis alpina*   \|  \| PRECYY \| SFROYY \| SLP25 \| SRADYY \| SWB \| TAVEYY \| TWI25SS \| \| --- \| --- \| --- \| --- \| --- \| --- \| --- \| --- \| \| ASPVAL \| 0.28 \| -0.60 \| 0.04 \| -0.13 \| -0.05 \| 0.11 \| 0.14 \| \| PRECYY \|  \| 0.31 \| -0.89 \| 0.28 \| -0.08 \| -0.60 \| -0.20 \| \| SFROYY \|  \|  \| -0.68 \| 0.11 \| -0.01 \| -0.85 \| 0.05 \| \| SLP25 \|  \|  \|  \| -0.35 \| -0.10 \| 0.85 \| -0.01 \| \| SRADYY \|  \|  \|  \|  \| 0.60 \| -0.04 \| -0.13 \| \| SWB \|  \|  \|  \|  \|  \| -0.03 \| 0.68 \| \| TAVEYY \|  \|  \|  \|  \|  \|  \| -0.25 \| |
| --- | --- | --- | --- | --- | --- | --- | --- | --- | --- | --- | --- | --- | --- | --- | --- | --- | --- | --- | --- | --- | --- | --- | --- | --- | --- | --- | --- | --- | --- | --- | --- | --- | --- | --- | --- | --- | --- | --- | --- | --- | --- | --- | --- | --- | --- | --- | --- | --- | --- | --- | --- | --- | --- | --- | --- | --- | --- | --- | --- | --- | --- | --- | --- | --- | --- | --- | --- | --- | --- | --- | --- | --- | --- | --- | --- | --- | --- | --- | --- | --- | --- | --- | --- | --- | --- | --- | --- | --- | --- | --- | --- | --- | --- | --- | --- | --- | --- | --- | --- | --- | --- | --- | --- | --- | --- | --- | --- | --- | --- | --- | --- | --- | --- | --- | --- | --- | --- | --- | --- | --- | --- | --- | --- | --- | --- | --- | --- | --- | --- |
| (c) *Arabidopsis halleri*   \|  \| PRECYY \| SFROYY \| SLP25 \| SRADYY \| SWB \| TAVEYY \| TWI25SS \| \| --- \| --- \| --- \| --- \| --- \| --- \| --- \| --- \| \| ASPVAL \| 0.22 \| -0.52 \| -0.96 \| -0.63 \| -0.32 \| 0.40 \| 0.52 \| \| PRECYY \|  \| 0.42 \| -0.28 \| 0.59 \| 0.70 \| -0.62 \| -0.55 \| \| SFROYY \|  \|  \| 0.50 \| 0.62 \| 0.45 \| -0.84 \| -0.52 \| \| SLP25 \|  \|  \|  \| 0.56 \| 0.12 \| -0.29 \| -0.38 \| \| SRADYY \|  \|  \|  \|  \| 0.80 \| -0.73 \| -0.87 \| \| SWB \|  \|  \|  \|  \|  \| -0.77 \| -0.90 \| \| TAVEYY \|  \|  \|  \|  \|  \|  \| 0.84 \| | (d) *Cardamine resedifolia*   \|  \| PRECYY \| SFROYY \| SLP25 \| SRADYY \| SWB \| TAVEYY \| TWI25SS \| \| --- \| --- \| --- \| --- \| --- \| --- \| --- \| --- \| \| ASPVAL \| 0.49 \| -0.42 \| 0.47 \| 0.86 \| 0.84 \| 0.31 \| 0.08 \| \| PRECYY \|  \| 0.16 \| -0.23 \| 0.78 \| 0.83 \| 0.01 \| -0.22 \| \| SFROYY \|  \|  \| -0.30 \| -0.16 \| -0.24 \| -0.91 \| -0.84 \| \| SLP25 \|  \|  \|  \| -0.03 \| 0.14 \| 0.12 \| -0.22 \| \| SRADYY \|  \|  \|  \|  \| 0.90 \| 0.17 \| 0.04 \| \| SWB \|  \|  \|  \|  \|  \| 0.21 \| 0.02 \| \| TAVEYY \|  \|  \|  \|  \|  \|  \| 0.80 \| |

**Table S3: Genetic diversity in the 18 study populations.** Shown is expected heterozygosity (*H*_e_) across all SNP loci for each population, and the mean and standard deviation for each species.

| **Species** | **Population** | ***H*_e_** | **Mean** | **Standard deviation** |
| --- | --- | --- | --- | --- |
| *Arabis alpina* | Aal05 | 0.095 | 0.138 | 0.040 |
|  | Aal18 | 0.104 |  |  |
|  | Aal27 | 0.149 |  |  |
|  | Aal31 | 0.112 |  |  |
|  | Aal34 | 0.186 |  |  |
|  | Aal36 | 0.183 |  |  |
| *Arabidopsis halleri* | Aha09 | 0.209 | 0.197 | 0.015 |
|  | Aha11 | 0.170 |  |  |
|  | Aha19 | 0.198 |  |  |
|  | Aha21 | 0.194 |  |  |
|  | Aha31 | 0.214 |  |  |
|  | AhaN3 | 0.198 |  |  |
| *Cardamine resedifolia* | Cre03 | 0.136 | 0.120 | 0.076 |
|  | Cre12 | 0.164 |  |  |
|  | Cre13 | 0.071 |  |  |
|  | Cre14 | 0.237 |  |  |
|  | Cre18 | 0.090 |  |  |
|  | Cre20 | 0.023 |  |  |

**Table S4: Genetic differentiation among the 6 study populations per species.** Shown is pairwise *F*_ST_ among populations of the same species using all SNPs.

| (a) *Arabis alpina*   \|  \| Aal18 \| Aal27 \| Aal31 \| Aal34 \| Aal36 \| \| --- \| --- \| --- \| --- \| --- \| --- \| \| Aal05 \| 0.451 \| 0.402 \| 0.460 \| 0.226 \| 0.261 \| \| Aal18 \|  \| 0.437 \| 0.493 \| 0.283 \| 0.286 \| \| Aal27 \|  \|  \| 0.361 \| 0.221 \| 0.228 \| \| Aal31 \|  \|  \|  \| 0.281 \| 0.281 \| \| Aal34 \|  \|  \|  \|  \| 0.089 \| | (b) *Arabidopsis halleri*   \|  \| Aha11 \| Aha19 \| Aha21 \| Aha31 \| AhaN3 \| \| --- \| --- \| --- \| --- \| --- \| --- \| \| Aha09 \| 0.119 \| 0.071 \| 0.067 \| 0.034 \| 0.050 \| \| Aha11 \|  \| 0.114 \| 0.134 \| 0.129 \| 0.100 \| \| Aha19 \|  \|  \| 0.080 \| 0.082 \| 0.050 \| \| Aha21 \|  \|  \|  \| 0.074 \| 0.054 \| \| Aha31 \|  \|  \|  \|  \| 0.059 \| |
| --- | --- | --- | --- | --- | --- | --- | --- | --- | --- | --- | --- | --- | --- | --- | --- | --- | --- | --- | --- | --- | --- | --- | --- | --- | --- | --- | --- | --- | --- | --- | --- | --- | --- | --- | --- | --- | --- | --- | --- | --- | --- | --- | --- | --- | --- | --- | --- | --- | --- | --- | --- | --- | --- | --- | --- | --- | --- | --- | --- | --- | --- | --- | --- | --- | --- | --- | --- | --- | --- | --- | --- | --- | --- |
| (c) *Cardamine resedifolia*   \|  \| Cre12 \| Cre13 \| Cre14 \| Cre18 \| Cre20 \| \| --- \| --- \| --- \| --- \| --- \| --- \| \| Cre03 \| 0.216 \| 0.689 \| 0.286 \| 0.663 \| 0.736 \| \| Cre12 \|  \| 0.662 \| 0.215 \| 0.623 \| 0.733 \| \| Cre13 \|  \|  \| 0.352 \| 0.273 \| 0.553 \| \| Cre14 \|  \|  \|  \| 0.314 \| 0.430 \| \| Cre18 \|  \|  \|  \|  \| 0.465 \| |  |

**Table S5: Comparison of population structure and environmental conditions.** Shown are the Pearson correlation coefficients for each environmental factor with the first two principal components (PC1 and PC2) of the principal component analyses shown in Figure S2. Aal *= Arabis alpina*; Aha *= Arabidopsis halleri*; Cre *= Cardamine resedifolia.* The percentage indicates the proportion of variance explained by each PC. **p* < 0.05, ***p* < 0.01

| **Environmental factor** | **Aal PC1**  (25.4%) | **Aal PC2**  (21.8%) | **Aha PC1**  (26.4%) | **Aha PC2**  (22.1%) | **Cre PC1**  (51.2%) | **Cre PC2**  (14.7%) |
| --- | --- | --- | --- | --- | --- | --- |
| Aspect | -0.29 | -0.24 | 0.09 | -0.62 | -0.39 | 0.41 |
| Precipitation | -0.73 | 0.53 | 0.13 | 0.46 | -0.15 | 0.78 |
| Frost | -0.23 | 0.65 | -0.43 | 0.5 | -0.1 | -0.36 |
| Slope | 0.42 | -0.76 | -0.31 | 0.55 | -0.15 | -0.11 |
| Radiation | -0.39 | -0.01 | 0.07 | 0.92** | -0.39 | 0.55 |
| Site water balance | 0.16 | 0.31 | 0.45 | 0.77 | -0.07 | 0.71 |
| Temperature | -0.08 | -0.77 | 0.17 | -0.74 | -0.08 | 0.57 |
| Topographic wetness index | 0.57 | 0.49 | -0.14 | -0.96* | 0.25 | 0.24 |

**Table S6: Overview of BayeScan outlier analyses.** Based on a false discovery rate of 5% in BayeScan. Numbers refer to outliers for directional selection only (positive ⍺_i_ value). Percentages indicate the proportion of genome-wide SNPs that are outliers (first row), and the proportion outlier SNPs without or with functional annotation (following rows).

|  | ***Arabis alpina*** | ***Arabidopsis halleri*** | ***Cardamine resedifolia*** |
| --- | --- | --- | --- |
| Number of outlier SNPs | 43,430 (1.27%) | 61,446 (1.80%) | 11,983 (0.44%) |
| Outlier SNPs without annotation | 37,879 (87.22%) | 46,864 (76.27%) | 10,804 (90.16%) |
| Outlier SNPs with annotation | 5,551 (12.78%) | 14,582 (23.73%) | 1,179 (9.84%) |
| Number of genes with annotated outlier SNPs | 1,883 | 3,864 | 670 |

**Table S7: Results of environmental association analyses using all SNPs.** Number of significant associations for all SNPs with eight environmental factors based on an FDR of 0.1% in LFMM.

|  |  |  |  |
| --- | --- | --- | --- |
| **Environmental factor** | ***Arabis alpina*** | ***Arabidopsis halleri*** | ***Cardamine resedifolia*** |
| Aspect | 11,548 | 38,647 | 147,942 |
| Precipitation | 282,855 | 71,485 | 122,452 |
| Frost | 67,735 | 43,738 | 88,888 |
| Slope | 249,753 | 42,478 | 132,422 |
| Radiation | 76,027 | 62,289 | 183,126 |
| Site water balance | 179,771 | 112,931 | 108,575 |
| Temperature | 173,829 | 67,280 | 66,301 |
| Topographic wetness index | 161,891 | 9,556 | 144,793 |
| Overall | 1,203,409 | 534,409 | 994,499 |
| % of significant associations | 4.40 | 1.64 | 4.55 |
| Number of associated SNPs | 591,325 | 248,483 | 431,389 |
| % of SNPs with significant associations | 17.31 | 7.29 | 15.78 |

**Table S8: Results of environmental association analyses for annotated SNPs**. Number of significant associations for all annotated SNPs with eight environmental factors based on an FDR of 0.1% in LFMM.

|  |  |  |  |
| --- | --- | --- | --- |
| **Environmental factor** | ***Arabis alpina*** | ***Arabidopsis halleri*** | ***Cardamine resedifolia*** |
| Aspect | 1,678 | 10,126 | 17,635 |
| Precipitation | 39,845 | 20,624 | 11,634 |
| Frost | 10,425 | 11,846 | 10,328 |
| Slope | 35,021 | 11,334 | 16,285 |
| Radiation | 10,461 | 16,876 | 17,735 |
| Site water balance | 24,885 | 32,181 | 11,961 |
| Temperature | 24,351 | 19,211 | 7,496 |
| Topographic wetness index | 22,696 | 27,295 | 16,946 |
| Number of annotated SNPs | 334,884 | 799,922 | 236,632 |
| Number associated annotated SNPs | 83,635 | 70,305 | 47,109 |
| % of annotated SNPs with significant associations | 24.97 | 8.78 | 19.91 |

**Table S9: Observed and expected number of shared genes with non-synonymous SNPs.** Shown are the total number of shared genes, the number of shared genes containing BayeScan outliers, and the number of shared genes associated with environmental factors. Proportion refers to the proportion of shared genes with genes containing non-synonymous (NS) SNPs. Expected indicates the mean number of genes with NS SNPs after random subsampling. The *p* value is based on the empirical distribution and shows the probability of a random draw to be above the observed value.

|  | **Shared genes** | **Observed number of genes with NS SNPs** | **Proportion (%)** | **Expected number of genes with NS SNPs** | ***p*** |
| --- | --- | --- | --- | --- | --- |
| All | 12,485 | 8,681 | 69.5 |  |  |
| BayScan outlier | 27 | 21 | 77.8 | 18.8 | 0.121 |
| Aspect | 47 | 38 | 80.9 | 32.8 | 0.027 |
| Precipitation | 586 | 502 | 85.7 | 407.5 | <0.001 |
| Frost | 123 | 107 | 87.0 | 85.5 | <0.001 |
| Slope | 462 | 399 | 86.4 | 321.2 | <0.001 |
| Radiation | 280 | 247 | 88.2 | 194.6 | <0.001 |
| Site water balance | 565 | 504 | 89.2 | 392.7 | <0.001 |
| Temperature | 283 | 247 | 87.3 | 196.9 | <0.001 |
| Topographic wetness index | 581 | 488 | 84.0 | 403.9 | <0.001 |

**Table S10: List of the 298 top candidate genes.** Included is a single gene containing non-synonymous (NS) BayeScan outlier SNPs and 297 genes containing NS SNPs associated to the same environmental factor in all three species. Given are TAIR ID, BayeScan outlier or associated environmental factor (1 = present, 0 = absent). ASPVAL = aspect; PRECYY = yearly precipitation sum; SFROYY = annual average of frost days; SLP25 = slope; SRADYY = yearly solar radiation; SWB = site water balance; TAVEYY = yearly temperature; TWI25SS = topographic wetness index (potential soil humidity). Also given is the short and full name of genes.

| **TAIR ID** | **Outlier** | **ASPVAL** | **PRECYY** | **SFROYY** | **SLP25** | **SRADYY** | **SWB** | **TAVEYY** | **TWI** | **Short name** | **Name** |
| --- | --- | --- | --- | --- | --- | --- | --- | --- | --- | --- | --- |
| AT1G03080 | 0 | 0 | 0 | 0 | 0 | 0 | 1 | 0 | 0 | *NET1D* | kinase interacting (KIP1-like) family protein |
| AT1G04790 | 0 | 0 | 0 | 0 | 1 | 0 | 0 | 0 | 0 |  | RING/U-box superfamily protein |
| AT1G04840 | 0 | 0 | 0 | 0 | 1 | 0 | 0 | 1 | 1 |  | Tetratricopeptide repeat (TPR)-like superfamily protein |
| AT1G04860 | 0 | 0 | 0 | 0 | 0 | 0 | 1 | 0 | 1 | *UBP2* | ubiquitin-specific protease 2 |
| AT1G04890 | 0 | 0 | 0 | 0 | 0 | 0 | 1 | 0 | 0 |  | zein-binding protein (Protein of unknown function, DUF593) |
| AT1G04920 | 0 | 0 | 1 | 0 | 0 | 0 | 1 | 1 | 1 | *SPS3F* | sucrose phosphate synthase 3F |
| AT1G04930 | 0 | 0 | 0 | 0 | 0 | 0 | 0 | 0 | 1 |  | hydroxyproline-rich glycoprotein family protein |
| AT1G04970 | 0 | 0 | 0 | 1 | 1 | 0 | 0 | 1 | 0 |  | lipid-binding serum glycoprotein family protein |
| AT1G05230 | 0 | 0 | 0 | 0 | 0 | 0 | 0 | 0 | 1 | *HDG2* | homeodomain GLABROUS 2 |
| AT1G05270 | 0 | 0 | 0 | 0 | 0 | 0 | 0 | 0 | 1 |  | TraB family protein |
| AT1G05280 | 1 | 0 | 0 | 0 | 0 | 0 | 0 | 0 | 0 |  | ERV-F (C)1 provirus ancestral Env polyprotein, putative (DUF604) |
| AT1G05380 | 0 | 0 | 1 | 0 | 0 | 1 | 1 | 0 | 0 |  | Acyl-CoA N-acyltransferase with RING/FYVE/PHD-type zinc finger protein |
| AT1G05385 | 0 | 0 | 0 | 0 | 0 | 1 | 1 | 0 | 0 | *LPA19* | photosystem II 11 kDa protein-like protein |
| AT1G05490 | 0 | 0 | 1 | 0 | 0 | 0 | 0 | 0 | 0 | *chr31* | chromatin remodeling 31 |
| AT1G05590 | 0 | 0 | 0 | 0 | 0 | 0 | 1 | 0 | 0 | *HEXO2* | beta-hexosaminidase 2 |
| AT1G05600 | 0 | 0 | 0 | 0 | 0 | 1 | 1 | 1 | 1 | *EMB311* | Tetratricopeptide repeat (TPR)-like superfamily protein |
| AT1G05700 | 0 | 0 | 0 | 0 | 0 | 1 | 0 | 0 | 0 |  | Leucine-rich repeat transmembrane protein kinase protein |
| AT1G06000 | 0 | 0 | 0 | 0 | 0 | 0 | 0 | 1 | 0 |  | UDP-Glycosyltransferase superfamily protein |
| AT1G06070 | 0 | 0 | 0 | 0 | 0 | 0 | 1 | 0 | 0 |  | Basic-leucine zipper (bZIP) transcription factor family protein |
| AT1G06970 | 0 | 0 | 0 | 0 | 0 | 0 | 0 | 0 | 1 | *CHX14* | cation/hydrogen exchanger 14 |
| AT1G07330 | 0 | 0 | 0 | 0 | 0 | 0 | 0 | 0 | 1 |  | dentin sialophosphoprotein |
| AT1G09600 | 0 | 0 | 0 | 0 | 0 | 0 | 0 | 0 | 1 |  | Protein kinase superfamily protein |
| AT1G10040 | 0 | 0 | 0 | 0 | 0 | 0 | 1 | 0 | 0 |  | alpha/beta-Hydrolases superfamily protein |
| AT1G10050 | 0 | 0 | 0 | 0 | 1 | 0 | 1 | 0 | 0 |  | glycosyl hydrolase family 10 protein / carbohydrate-binding domain-containing protein |
| AT1G11300 | 0 | 0 | 0 | 0 | 0 | 0 | 0 | 0 | 1 |  | G-type lectin S-receptor-like Serine/Threonine-kinase |
| AT1G11740 | 0 | 0 | 0 | 0 | 0 | 0 | 0 | 0 | 1 |  | ankyrin repeat family protein |
| AT1G12970 | 0 | 0 | 0 | 0 | 0 | 0 | 0 | 0 | 1 | *PIRL3* | plant intracellular ras group-related LRR 3 |
| AT1G14040 | 0 | 0 | 0 | 0 | 0 | 0 | 0 | 0 | 1 |  | EXS (ERD1/XPR1/SYG1) family protein |
| AT1G14470 | 0 | 0 | 0 | 0 | 1 | 0 | 0 | 0 | 1 |  | Pentatricopeptide repeat (PPR) superfamily protein |
| AT1G14520 | 0 | 0 | 0 | 0 | 0 | 0 | 0 | 0 | 1 | *MIOX1* | myo-inositol oxygenase 1 |
| AT1G14686 | 0 | 0 | 0 | 0 | 0 | 0 | 0 | 0 | 1 |  | ENTH/ANTH/VHS superfamily protein |
| AT1G14910 | 0 | 0 | 0 | 0 | 0 | 0 | 0 | 0 | 1 |  | ENTH/ANTH/VHS superfamily protein |
| AT1G15290 | 0 | 0 | 0 | 0 | 0 | 0 | 1 | 1 | 1 |  | Tetratricopeptide repeat (TPR)-like superfamily protein |
| AT1G15320 | 0 | 0 | 0 | 0 | 0 | 0 | 0 | 0 | 1 |  | seed dormancy control protein |
| AT1G16260 | 0 | 0 | 0 | 0 | 0 | 0 | 1 | 0 | 0 |  | Wall-associated kinase family protein |
| AT1G19940 | 0 | 0 | 0 | 0 | 0 | 0 | 0 | 0 | 1 | *GH9B5* | glycosyl hydrolase 9B5 |
| AT1G20150 | 0 | 0 | 0 | 0 | 1 | 0 | 0 | 0 | 1 |  | Subtilisin-like serine endopeptidase family protein |
| AT1G20180 | 0 | 0 | 0 | 0 | 0 | 1 | 0 | 0 | 0 |  | transmembrane protein (DUF677) |
| AT1G22100 | 0 | 0 | 0 | 0 | 0 | 0 | 0 | 0 | 1 |  | Inositol-pentakisphosphate 2-kinase family protein |
| AT1G22860 | 0 | 0 | 0 | 0 | 0 | 0 | 1 | 0 | 0 |  | Vacuolar sorting protein 39 |
| AT1G23200 | 0 | 0 | 0 | 0 | 0 | 0 | 1 | 0 | 0 |  | Plant invertase/pectin methylesterase inhibitor superfamily |
| AT1G23230 | 0 | 0 | 0 | 0 | 0 | 0 | 0 | 0 | 1 |  | mediator of RNA polymerase II transcription subunit |
| AT1G23400 | 0 | 0 | 0 | 1 | 1 | 0 | 0 | 0 | 0 | *CAF2* | RNA-binding CRS1 / YhbY (CRM) domain-containing protein |
| AT1G23890 | 0 | 0 | 0 | 0 | 0 | 0 | 0 | 0 | 1 |  | NHL domain-containing protein |
| AT1G24420 | 0 | 0 | 0 | 0 | 0 | 0 | 0 | 0 | 1 |  | HXXXD-type acyl-transferase family protein |
| AT1G25390 | 0 | 0 | 1 | 0 | 0 | 0 | 0 | 1 | 0 |  | Protein kinase superfamily protein |
| AT1G27595 | 0 | 0 | 0 | 0 | 1 | 0 | 0 | 0 | 0 |  | symplekin |
| AT1G27752 | 0 | 0 | 0 | 0 | 0 | 0 | 0 | 1 | 0 |  | Ubiquitin system component Cue protein |
| AT1G28530 | 0 | 0 | 0 | 0 | 0 | 0 | 1 | 0 | 0 |  | deneddylase |
| AT1G32375 | 0 | 0 | 0 | 0 | 0 | 0 | 0 | 1 | 0 |  | F-box/RNI-like/FBD-like domains-containing protein |
| AT1G32380 | 0 | 0 | 0 | 1 | 0 | 0 | 0 | 0 | 0 | *PRS2* | phosphoribosyl pyrophosphate (PRPP) synthase 2 |
| AT1G32440 | 0 | 0 | 1 | 0 | 0 | 0 | 0 | 0 | 0 | *PKp3* | plastidial pyruvate kinase 3 |
| AT1G33590 | 0 | 0 | 1 | 0 | 0 | 0 | 1 | 0 | 0 |  | Leucine-rich repeat (LRR) family protein |
| AT1G34110 | 0 | 0 | 0 | 0 | 0 | 0 | 1 | 0 | 0 |  | Leucine-rich receptor-like protein kinase family protein |
| AT1G34320 | 0 | 0 | 0 | 0 | 1 | 0 | 0 | 0 | 0 |  | Ikzf5 (DUF668) |
| AT1G34355 | 0 | 0 | 1 | 0 | 0 | 0 | 1 | 0 | 0 | *PS1* | forkhead-associated (FHA) domain-containing protein |
| AT1G34580 | 0 | 0 | 1 | 0 | 0 | 0 | 0 | 0 | 0 |  | Major facilitator superfamily protein |
| AT1G48300 | 0 | 0 | 0 | 0 | 0 | 0 | 0 | 0 | 1 | *DGAT3* | diacylglycerol acyltransferase |
| AT1G49790 | 0 | 0 | 0 | 0 | 1 | 0 | 0 | 0 | 0 |  | F-box associated ubiquitination effector family protein |
| AT1G53420 | 0 | 0 | 1 | 0 | 0 | 0 | 0 | 0 | 0 |  | Leucine-rich repeat transmembrane protein kinase |
| AT1G54920 | 0 | 0 | 0 | 0 | 0 | 0 | 1 | 0 | 0 |  | hypothetical protein |
| AT1G54990 | 0 | 0 | 0 | 0 | 0 | 0 | 0 | 0 | 1 | *AXR4* | alpha/beta-Hydrolases superfamily protein |
| AT1G55180 | 0 | 0 | 1 | 0 | 0 | 0 | 0 | 0 | 0 | *PLDEPSILON* | phospholipase D alpha 4 |
| AT1G55370 | 0 | 0 | 0 | 0 | 0 | 0 | 0 | 0 | 1 | *NDF5* | NDH-dependent cyclic electron flow 5 |
| AT1G55860 | 0 | 0 | 0 | 0 | 0 | 0 | 1 | 0 | 0 | *UPL1* | LOW protein: E3 ubiquitin ligase-like protein |
| AT1G56570 | 0 | 0 | 0 | 0 | 0 | 0 | 1 | 0 | 1 | *PGN* | Tetratricopeptide repeat (TPR)-like superfamily protein |
| AT1G56690 | 0 | 0 | 0 | 0 | 0 | 0 | 1 | 0 | 0 |  | Pentatricopeptide repeat (PPR) superfamily protein |
| AT1G58250 | 0 | 0 | 0 | 0 | 1 | 0 | 1 | 0 | 0 | *SAB* | HYPERSENSITIVE TO PI STARVATION 4 |
| AT1G58370 | 0 | 0 | 0 | 0 | 0 | 0 | 1 | 0 | 0 | *RXF12* | glycosyl hydrolase family 10 protein / carbohydrate-binding domain-containing protein |
| AT1G59720 | 0 | 0 | 0 | 0 | 0 | 0 | 0 | 1 | 0 | *CRR28* | Tetratricopeptide repeat (TPR)-like superfamily protein |
| AT1G60560 | 0 | 0 | 1 | 0 | 0 | 0 | 0 | 0 | 0 |  | SWIM zinc finger family protein |
| AT1G60700 | 0 | 0 | 1 | 0 | 0 | 0 | 0 | 0 | 0 |  | SMAD/FHA domain-containing protein |
| AT1G60930 | 0 | 0 | 1 | 0 | 0 | 0 | 1 | 0 | 0 | *RECQ4B* | RECQ helicase L4B |
| AT1G61100 | 0 | 0 | 1 | 0 | 0 | 0 | 0 | 0 | 0 |  | disease resistance protein (TIR class) |
| AT1G61870 | 0 | 0 | 0 | 0 | 1 | 0 | 0 | 0 | 0 | *PPR336* | pentatricopeptide repeat 336 |
| AT1G62440 | 0 | 0 | 0 | 0 | 0 | 0 | 1 | 0 | 0 | *LRX2* | leucine-rich repeat/extensin 2 |
| AT1G62480 | 0 | 0 | 0 | 1 | 0 | 0 | 0 | 1 | 0 |  | Vacuolar calcium-binding protein-like protein |
| AT1G62710 | 0 | 0 | 0 | 0 | 0 | 0 | 1 | 0 | 1 | *BETA-VPE* | beta vacuolar processing enzyme |
| AT1G62720 | 0 | 0 | 0 | 0 | 0 | 1 | 1 | 0 | 1 | *NG1* | Pentatricopeptide repeat (PPR-like) superfamily protein |
| AT1G62850 | 0 | 0 | 0 | 1 | 0 | 0 | 0 | 0 | 0 |  | Class I peptide chain release factor |
| AT1G62960 | 0 | 0 | 0 | 0 | 1 | 0 | 0 | 0 | 0 | *ACS1* | ACC synthase 10 |
| AT1G63940 | 0 | 0 | 0 | 0 | 0 | 0 | 1 | 0 | 0 | *MDAR6* | monodehydroascorbate reductase 6 |
| AT1G64050 | 0 | 0 | 0 | 0 | 0 | 0 | 0 | 0 | 1 |  | hypothetical protein |
| AT1G64110 | 0 | 0 | 1 | 0 | 0 | 0 | 0 | 1 | 0 | *DAA1* | P-loop containing nucleoside triphosphate hydrolases superfamily protein |
| AT1G64170 | 0 | 0 | 0 | 0 | 0 | 0 | 0 | 1 | 0 | *CHX16* | cation/H+ exchanger 16 |
| AT1G64280 | 0 | 0 | 0 | 0 | 0 | 0 | 1 | 0 | 0 | *NPR1* | regulatory protein (NPR1) |
| AT1G64385 | 0 | 0 | 1 | 0 | 0 | 0 | 0 | 0 | 0 |  | transmembrane protein |
| AT1G64630 | 0 | 0 | 0 | 0 | 0 | 0 | 1 | 0 | 0 | *WNK1* | with no lysine (K) kinase 10 |
| AT1G65070 | 0 | 0 | 0 | 0 | 1 | 0 | 0 | 0 | 0 |  | DNA mismatch repair protein MutS, type 2 |
| AT1G65900 | 0 | 0 | 0 | 0 | 1 | 0 | 0 | 0 | 0 |  | plant/protein |
| AT1G66110 | 0 | 0 | 0 | 0 | 0 | 0 | 0 | 0 | 1 |  | hypothetical protein (DUF577) |
| AT1G66830 | 0 | 0 | 0 | 0 | 1 | 0 | 0 | 0 | 0 |  | Leucine-rich repeat protein kinase family protein |
| AT1G67120 | 0 | 1 | 0 | 0 | 0 | 0 | 0 | 0 | 0 |  | midasin-like protein |
| AT1G68720 | 0 | 0 | 0 | 0 | 0 | 1 | 0 | 0 | 0 | *TADA* | tRNA arginine adenosine deaminase |
| AT1G68890 | 0 | 0 | 1 | 0 | 0 | 0 | 0 | 0 | 0 | *PHYLLO* | 2-oxoglutarate decarboxylase/hydro-lyase/magnesium ion-binding protein |
| AT1G68990 | 0 | 0 | 1 | 0 | 0 | 0 | 0 | 0 | 0 | *MGP3* | male gametophyte defective 3 |
| AT1G69260 | 0 | 0 | 0 | 0 | 0 | 0 | 1 | 0 | 0 | *AFP1* | ABI five binding protein |
| AT1G70200 | 0 | 0 | 1 | 0 | 0 | 1 | 1 | 0 | 0 |  | RNA-binding (RRM/RBD/RNP motifs) family protein |
| AT1G70650 | 0 | 0 | 0 | 0 | 0 | 0 | 0 | 0 | 1 |  | Ran BP2/NZF zinc finger-like superfamily protein |
| AT1G70750 | 0 | 0 | 0 | 0 | 0 | 0 | 0 | 0 | 1 |  | myosin-binding protein (Protein of unknown function, DUF593) |
| AT1G71150 | 0 | 0 | 1 | 0 | 0 | 0 | 0 | 0 | 0 |  | cyclin-D1-binding protein |
| AT1G71696 | 0 | 0 | 1 | 0 | 0 | 0 | 0 | 0 | 0 | *SOL1* | carboxypeptidase D |
| AT1G71770 | 0 | 0 | 1 | 0 | 0 | 0 | 0 | 0 | 0 | *PAB5* | poly(A)-binding protein 5 |
| AT1G74680 | 0 | 0 | 0 | 0 | 0 | 1 | 0 | 0 | 0 |  | Exostosin family protein |
| AT1G75100 | 0 | 0 | 0 | 0 | 1 | 0 | 0 | 0 | 0 | *JAC1* | J-domain protein required for chloroplast accumulation response 1 |
| AT1G75640 | 0 | 0 | 0 | 0 | 1 | 0 | 0 | 0 | 0 |  | Leucine-rich receptor-like protein kinase family protein |
| AT1G76700 | 0 | 0 | 0 | 0 | 0 | 0 | 1 | 0 | 0 |  | DNAJ heat shock N-terminal domain-containing protein |
| AT1G77800 | 0 | 0 | 0 | 0 | 0 | 0 | 0 | 0 | 1 |  | PHD finger family protein |
| AT1G78060 | 0 | 0 | 0 | 0 | 0 | 0 | 0 | 1 | 0 |  | Glycosyl hydrolase family protein |
| AT1G79620 | 0 | 0 | 0 | 0 | 0 | 1 | 0 | 0 | 0 |  | Leucine-rich repeat protein kinase family protein |
| AT1G80530 | 0 | 0 | 0 | 0 | 0 | 0 | 1 | 0 | 0 |  | Major facilitator superfamily protein |
| AT1G80550 | 0 | 0 | 0 | 0 | 0 | 0 | 1 | 0 | 1 |  | Pentatricopeptide repeat (PPR) superfamily protein |
| AT1G80570 | 0 | 0 | 0 | 0 | 0 | 0 | 1 | 0 | 1 |  | RNI-like superfamily protein |
| AT1G80640 | 0 | 0 | 0 | 0 | 0 | 0 | 1 | 0 | 0 |  | Protein kinase superfamily protein |
| AT1G80680 | 0 | 0 | 0 | 0 | 0 | 0 | 1 | 0 | 0 | *SAR3* | SUPPRESSOR OF AUXIN RESISTANCE 3 |
| AT2G03140 | 0 | 0 | 0 | 0 | 1 | 0 | 0 | 0 | 0 |  | alpha/beta-Hydrolases superfamily protein |
| AT2G03880 | 0 | 0 | 1 | 0 | 1 | 0 | 0 | 0 | 1 | *REME1* | Pentatricopeptide repeat (PPR) superfamily protein |
| AT2G04235 | 0 | 0 | 0 | 0 | 1 | 0 | 0 | 0 | 0 |  | hypothetical protein |
| AT2G04660 | 0 | 0 | 1 | 1 | 0 | 0 | 0 | 0 | 0 | *APC2* | anaphase-promoting complex/cyclosome 2 |
| AT2G04845 | 0 | 0 | 0 | 0 | 0 | 0 | 0 | 0 | 1 |  | Acyl-CoA N-acyltransferases (NAT) superfamily protein |
| AT2G05120 | 0 | 0 | 0 | 0 | 1 | 0 | 0 | 0 | 1 |  | Nucleoporin, Nup133/Nup155-like protein |
| AT2G12462 | 0 | 0 | 1 | 0 | 0 | 0 | 1 | 0 | 0 |  | sterile alpha motif (SAM) domain protein |
| AT2G14540 | 0 | 0 | 0 | 1 | 0 | 0 | 0 | 0 | 0 | *SRP2* | serpin 2 |
| AT2G15690 | 0 | 0 | 1 | 0 | 0 | 0 | 0 | 0 | 0 |  | Tetratricopeptide repeat (TPR)-like superfamily protein |
| AT2G16250 | 0 | 0 | 1 | 0 | 0 | 0 | 0 | 0 | 0 |  | Leucine-rich repeat protein kinase family protein |
| AT2G16440 | 0 | 0 | 1 | 0 | 0 | 0 | 0 | 0 | 0 | *MCM4* | Minichromosome maintenance (MCM2/3/5) family protein |
| AT2G19270 | 0 | 0 | 0 | 0 | 0 | 0 | 1 | 0 | 0 |  | mitotic checkpoint protein PRCC-carboxy-term protein |
| AT2G19640 | 0 | 0 | 0 | 0 | 0 | 0 | 0 | 0 | 1 | *ASHR2* | ASH1-related protein 2 |
| AT2G19710 | 0 | 0 | 1 | 1 | 0 | 0 | 1 | 1 | 1 |  | Regulator of Vps4 activity in the MVB pathway protein |
| AT2G20960 | 0 | 0 | 0 | 0 | 1 | 0 | 0 | 0 | 0 | *pEARLI4* | phospholipase-like protein (PEARLI 4) family protein |
| AT2G23360 | 0 | 0 | 1 | 0 | 0 | 0 | 0 | 1 | 0 |  | filament-like protein (DUF869) |
| AT2G25320 | 0 | 0 | 0 | 1 | 1 | 0 | 0 | 0 | 0 |  | TRAF-like family protein |
| AT2G25710 | 0 | 0 | 0 | 0 | 1 | 0 | 0 | 0 | 0 | *HCS1* | holocarboxylase synthase 1 |
| AT2G25740 | 0 | 0 | 0 | 0 | 0 | 0 | 0 | 0 | 1 |  | ATP-dependent protease La (LON) domain protein |
| AT2G26170 | 0 | 0 | 0 | 0 | 0 | 0 | 1 | 0 | 0 | *CYP711A1* | cytochrome P450, family 711, subfamily A, polypeptide 1 |
| AT2G26270 | 0 | 0 | 0 | 0 | 0 | 0 | 0 | 0 | 1 |  | BRCT domain DNA repair protein |
| AT2G26290 | 0 | 0 | 0 | 0 | 0 | 0 | 0 | 1 | 0 | *ARSK1* | root-specific kinase 1 |
| AT2G26460 | 0 | 0 | 0 | 0 | 1 | 0 | 0 | 0 | 0 | *SMU2* | RED family protein |
| AT2G28130 | 0 | 0 | 0 | 0 | 1 | 0 | 0 | 1 | 0 |  | actin protein 2/3 complex subunit-like protein |
| AT2G29210 | 0 | 0 | 0 | 0 | 0 | 0 | 0 | 1 | 0 |  | splicing factor PWI domain-containing protein |
| AT2G30210 | 0 | 0 | 0 | 0 | 0 | 0 | 1 | 0 | 0 | *LAC3* | laccase 3 |
| AT2G30290 | 0 | 0 | 0 | 0 | 0 | 0 | 0 | 0 | 1 | *VSR2* | VACUOLAR SORTING RECEPTOR 2 |
| AT2G30490 | 0 | 0 | 0 | 0 | 0 | 0 | 1 | 0 | 0 | *C4H* | cinnamate-4-hydroxylase |
| AT2G31560 | 0 | 0 | 0 | 1 | 0 | 0 | 0 | 0 | 0 |  | signal transducer/transcription protein, putative (DUF1685) |
| AT2G31900 | 0 | 0 | 0 | 1 | 0 | 0 | 0 | 0 | 0 | *XIF* | myosin-like protein XIF |
| AT2G31920 | 0 | 0 | 0 | 0 | 0 | 0 | 1 | 0 | 0 |  | elongation factor 1-delta, putative (DUF936) |
| AT2G32240 | 0 | 0 | 0 | 0 | 0 | 0 | 0 | 0 | 1 |  | early endosome antigen |
| AT2G32415 | 0 | 0 | 1 | 0 | 0 | 0 | 0 | 0 | 0 |  | Polynucleotidyl transferase, ribonuclease H fold protein with HRDC domain-containing protein |
| AT2G33435 | 0 | 0 | 0 | 0 | 0 | 0 | 0 | 0 | 1 |  | RNA-binding (RRM/RBD/RNP motifs) family protein |
| AT2G34370 | 0 | 0 | 0 | 0 | 0 | 1 | 0 | 0 | 0 |  | Pentatricopeptide repeat (PPR) superfamily protein |
| AT2G34780 | 0 | 0 | 0 | 0 | 0 | 1 | 0 | 0 | 0 | *MEE22* | maternal effect embryo arrest 22 |
| AT2G36350 | 0 | 0 | 0 | 0 | 1 | 0 | 0 | 0 | 0 |  | Protein kinase superfamily protein |
| AT2G36480 | 0 | 0 | 0 | 0 | 0 | 0 | 0 | 0 | 1 |  | pre-mRNA cleavage complex 2 Pcf11-like protein |
| AT2G37290 | 0 | 0 | 0 | 0 | 0 | 0 | 1 | 0 | 0 |  | Ypt/Rab-GAP domain of gyp1p superfamily protein |
| AT2G37550 | 0 | 0 | 1 | 0 | 0 | 0 | 0 | 0 | 0 | *AGD7* | ARF-GAP domain 7 |
| AT2G38995 | 0 | 0 | 0 | 0 | 0 | 0 | 0 | 0 | 1 |  | O-acyltransferase (WSD1-like) family protein |
| AT2G41120 | 0 | 0 | 1 | 0 | 0 | 0 | 0 | 0 | 0 |  | DUF309 domain protein |
| AT2G41880 | 0 | 0 | 0 | 0 | 0 | 0 | 0 | 0 | 1 | *GK-1* | guanylate kinase 1 |
| AT2G41890 | 0 | 0 | 0 | 0 | 0 | 0 | 0 | 0 | 1 |  | curculin-like (mannose-binding) lectin family protein / PAN domain-containing protein |
| AT3G02010 | 0 | 0 | 0 | 0 | 0 | 0 | 1 | 0 | 0 |  | Pentatricopeptide repeat (PPR) superfamily protein |
| AT3G05370 | 0 | 0 | 1 | 0 | 0 | 0 | 0 | 0 | 0 | *RLP31* | receptor like protein 31 |
| AT3G07040 | 0 | 0 | 0 | 0 | 0 | 0 | 0 | 0 | 1 | *RPM1* | NB-ARC domain-containing disease resistance protein |
| AT3G10340 | 0 | 0 | 0 | 0 | 0 | 0 | 0 | 0 | 1 | *PAL4* | phenylalanine ammonia-lyase 4 |
| AT3G11490 | 0 | 0 | 1 | 0 | 0 | 0 | 0 | 0 | 0 |  | rac GTPase activating protein |
| AT3G12000 | 0 | 0 | 0 | 0 | 0 | 0 | 1 | 1 | 0 |  | S-locus related protein SLR1, putative (S1) |
| AT3G13770 | 0 | 0 | 0 | 0 | 0 | 0 | 0 | 0 | 1 |  | Pentatricopeptide repeat (PPR) superfamily protein |
| AT3G14172 | 0 | 0 | 0 | 0 | 0 | 0 | 1 | 0 | 1 |  | GPI-anchored adhesin-like protein |
| AT3G15550 | 0 | 0 | 1 | 0 | 0 | 0 | 0 | 0 | 0 |  | trichohyalin |
| AT3G16890 | 0 | 0 | 0 | 0 | 0 | 0 | 1 | 0 | 0 | *PPR4* | pentatricopeptide (PPR) domain protein 40 |
| AT3G16910 | 0 | 0 | 0 | 0 | 1 | 0 | 0 | 0 | 0 | *AAE7* | acyl-activating enzyme 7 |
| AT3G17040 | 0 | 1 | 0 | 0 | 0 | 0 | 0 | 0 | 0 | *HCF17* | high chlorophyll fluorescent 107 |
| AT3G17360 | 0 | 0 | 0 | 0 | 0 | 0 | 1 | 0 | 0 | *POK1* | phragmoplast orienting kinesin 1 |
| AT3G17740 | 0 | 0 | 0 | 0 | 0 | 1 | 1 | 0 | 0 |  | hypothetical protein |
| AT3G19190 | 0 | 0 | 1 | 0 | 0 | 0 | 0 | 0 | 0 | *ATG2* | autophagy 2 |
| AT3G19230 | 0 | 0 | 0 | 0 | 0 | 0 | 0 | 0 | 1 |  | Leucine-rich repeat (LRR) family protein |
| AT3G20200 | 0 | 0 | 1 | 0 | 0 | 0 | 0 | 0 | 0 |  | kinase with adenine nucleotide alpha hydrolases-like domain-containing protein |
| AT3G20475 | 0 | 0 | 1 | 0 | 0 | 0 | 0 | 0 | 0 | *MSH5* | MUTS-homologue 5 |
| AT3G20540 | 0 | 0 | 1 | 0 | 0 | 0 | 0 | 0 | 0 | *POLGAMMA1* | polymerase gamma 1 |
| AT3G24630 | 0 | 0 | 1 | 0 | 0 | 0 | 1 | 0 | 0 | *TRM34* | hypothetical protein |
| AT3G26380 | 0 | 0 | 0 | 0 | 0 | 0 | 0 | 0 | 1 |  | Melibiase family protein |
| AT3G27700 | 0 | 0 | 0 | 0 | 0 | 0 | 0 | 1 | 0 |  | zinc finger (CCCH-type) family protein / RNA recognition motif (RRM)-containing protein |
| AT3G28030 | 0 | 0 | 0 | 1 | 0 | 0 | 0 | 0 | 0 | *UVH3* | 5'-3' exonuclease family protein |
| AT3G44910 | 0 | 0 | 0 | 0 | 1 | 0 | 0 | 0 | 0 | *CHX12* | cation/H+ exchanger 12 |
| AT3G46330 | 0 | 0 | 0 | 1 | 0 | 0 | 0 | 1 | 1 | *MEE39* | Leucine-rich repeat protein kinase family protein |
| AT3G46960 | 0 | 0 | 0 | 0 | 0 | 0 | 1 | 0 | 0 |  | RNA helicase, ATP-dependent, SK12/DOB1 protein |
| AT3G46990 | 0 | 0 | 0 | 0 | 0 | 1 | 1 | 0 | 0 |  | DUF740 family protein, putative (DUF740) |
| AT3G47580 | 0 | 0 | 0 | 0 | 1 | 0 | 0 | 0 | 0 |  | Leucine-rich repeat protein kinase family protein |
| AT3G48190 | 0 | 0 | 0 | 0 | 0 | 0 | 1 | 0 | 1 | *ATM* | Serine/Threonine-kinase ATM-like protein |
| AT3G48200 | 0 | 0 | 0 | 0 | 0 | 0 | 0 | 0 | 1 |  | transmembrane protein |
| AT3G49670 | 0 | 0 | 0 | 0 | 1 | 0 | 1 | 1 | 0 | *BAM2* | Leucine-rich receptor-like protein kinase family protein |
| AT3G52070 | 0 | 0 | 1 | 0 | 0 | 1 | 0 | 0 | 0 |  | RNA/RNP complex-1-interacting phosphatase |
| AT3G55480 | 0 | 0 | 0 | 0 | 0 | 0 | 1 | 0 | 0 | *PAT2* | protein affected traf%26#64257%3Bcking 2 |
| AT3G56320 | 0 | 0 | 1 | 0 | 0 | 0 | 0 | 0 | 0 |  | PAP/OAS1 substrate-binding domain superfamily |
| AT3G56750 | 0 | 0 | 0 | 0 | 0 | 0 | 0 | 0 | 1 |  | plant/protein |
| AT3G57780 | 0 | 0 | 0 | 1 | 1 | 0 | 0 | 0 | 0 |  | nucleolar-like protein |
| AT3G57980 | 0 | 0 | 0 | 1 | 0 | 0 | 0 | 1 | 0 |  | DNA-binding bromodomain-containing protein |
| AT3G58160 | 0 | 0 | 0 | 0 | 1 | 0 | 0 | 0 | 0 | *XIJ* | P-loop containing nucleoside triphosphate hydrolases superfamily protein |
| AT3G59140 | 0 | 0 | 1 | 0 | 0 | 0 | 0 | 0 | 0 | *ABCC1* | multidrug resistance-associated protein 14 |
| AT3G60050 | 0 | 0 | 0 | 0 | 0 | 1 | 0 | 0 | 0 |  | Pentatricopeptide repeat (PPR) superfamily protein |
| AT3G60680 | 0 | 0 | 0 | 0 | 1 | 0 | 0 | 0 | 0 |  | DUF641 family protein (DUF641) |
| AT3G60740 | 0 | 0 | 0 | 0 | 0 | 1 | 0 | 0 | 1 | *TTN1* | ARM repeat superfamily protein |
| AT3G60920 | 0 | 0 | 0 | 0 | 0 | 1 | 0 | 0 | 0 |  | beige/BEACH domain protein |
| AT3G61180 | 0 | 0 | 0 | 0 | 1 | 0 | 0 | 0 | 0 |  | RING/U-box superfamily protein |
| AT3G61670 | 0 | 0 | 1 | 0 | 0 | 0 | 0 | 0 | 0 |  | extra-large G-like protein, putative (DUF3133) |
| AT4G00060 | 0 | 0 | 0 | 0 | 0 | 0 | 0 | 0 | 1 | *MEE44* | Nucleotidyltransferase family protein |
| AT4G00350 | 0 | 0 | 0 | 0 | 1 | 0 | 0 | 0 | 0 |  | MATE efflux family protein |
| AT4G00450 | 0 | 0 | 1 | 0 | 0 | 0 | 0 | 0 | 0 | *CCT* | RNA polymerase II transcription mediator |
| AT4G01400 | 0 | 0 | 1 | 0 | 0 | 0 | 0 | 0 | 0 |  | oligomeric golgi complex subunit-like protein |
| AT4G02660 | 0 | 0 | 0 | 0 | 1 | 1 | 0 | 0 | 0 |  | Beige/BEACH and WD40 domain-containing protein |
| AT4G03130 | 0 | 0 | 0 | 0 | 0 | 0 | 1 | 0 | 0 |  | BRCT domain-containing DNA repair protein |
| AT4G03820 | 0 | 0 | 1 | 0 | 0 | 0 | 0 | 0 | 0 |  | transmembrane protein, putative (DUF3537) |
| AT4G04980 | 0 | 0 | 0 | 1 | 0 | 0 | 0 | 0 | 0 |  | hypothetical protein |
| AT4G11130 | 0 | 0 | 0 | 0 | 0 | 0 | 0 | 1 | 1 | *RDR2* | RNA-dependent RNA polymerase 2 |
| AT4G11160 | 0 | 0 | 0 | 0 | 1 | 0 | 0 | 0 | 0 |  | Translation initiation factor 2, small GTP-binding protein |
| AT4G12000 | 0 | 0 | 1 | 0 | 0 | 0 | 0 | 0 | 0 |  | SNARE associated Golgi protein family |
| AT4G12750 | 0 | 0 | 1 | 0 | 0 | 0 | 0 | 0 | 1 |  | Homeodomain-like transcriptional regulator |
| AT4G13840 | 0 | 0 | 0 | 0 | 0 | 0 | 0 | 0 | 1 |  | HXXXD-type acyl-transferase family protein |
| AT4G15730 | 0 | 0 | 0 | 0 | 0 | 0 | 0 | 1 | 0 |  | CW-type Zinc Finger |
| AT4G15980 | 0 | 0 | 0 | 0 | 1 | 0 | 0 | 0 | 0 |  | Plant invertase/pectin methylesterase inhibitor superfamily |
| AT4G17570 | 0 | 0 | 0 | 0 | 0 | 0 | 1 | 0 | 0 | *GATA26* | GATA transcription factor 26 |
| AT4G17610 | 0 | 0 | 0 | 0 | 0 | 0 | 1 | 0 | 0 |  | tRNA/rRNA methyltransferase (SpoU) family protein |
| AT4G17860 | 0 | 0 | 0 | 0 | 0 | 0 | 1 | 0 | 0 |  | carboxyl-terminal proteinase-like protein, putative (DUF239) |
| AT4G18010 | 0 | 0 | 0 | 0 | 1 | 0 | 0 | 0 | 0 | *IP5PII* | myo-inositol polyphosphate 5-phosphatase 2 |
| AT4G19191 | 0 | 0 | 0 | 0 | 0 | 0 | 1 | 0 | 0 |  | Tetratricopeptide repeat (TPR)-like superfamily protein |
| AT4G19610 | 0 | 0 | 0 | 0 | 0 | 0 | 0 | 0 | 1 |  | nucleotide/nucleic acid binding protein |
| AT4G20060 | 0 | 0 | 1 | 0 | 0 | 1 | 0 | 0 | 1 | *EMB1895* | ARM repeat superfamily protein |
| AT4G20130 | 0 | 0 | 0 | 0 | 0 | 0 | 1 | 0 | 0 | *PTAC14* | plastid transcriptionally active 14 |
| AT4G20160 | 0 | 0 | 0 | 0 | 0 | 0 | 1 | 0 | 1 |  | golgin family A protein |
| AT4G23000 | 0 | 0 | 1 | 0 | 0 | 0 | 0 | 0 | 0 |  | Calcineurin-like metallo-phosphoesterase superfamily protein |
| AT4G23290 | 0 | 0 | 0 | 0 | 0 | 0 | 1 | 0 | 0 | *CRK21* | cysteine-rich RLK (RECEPTOR-like protein kinase) 21 |
| AT4G24900 | 0 | 0 | 1 | 0 | 1 | 0 | 0 | 0 | 0 | *TTL* | coiled coil protein |
| AT4G26660 | 0 | 0 | 1 | 0 | 0 | 0 | 0 | 0 | 0 |  | kinesin-like protein |
| AT4G29440 | 0 | 0 | 0 | 0 | 0 | 0 | 1 | 0 | 1 |  | Regulator of Vps4 activity in the MVB pathway protein |
| AT4G34280 | 0 | 0 | 0 | 0 | 1 | 0 | 0 | 0 | 0 |  | transducin family protein / WD-40 repeat family protein |
| AT4G35520 | 0 | 0 | 1 | 0 | 0 | 0 | 0 | 0 | 0 | *MLH3* | MUTL protein homolog 3 |
| AT4G36090 | 0 | 0 | 0 | 0 | 0 | 0 | 0 | 1 | 0 |  | oxidoreductase, 2OG-Fe(II) oxygenase family protein |
| AT4G36210 | 0 | 0 | 0 | 0 | 0 | 0 | 0 | 1 | 0 |  | transmembrane/coiled-coil protein (DUF726) |
| AT4G36430 | 0 | 0 | 1 | 0 | 0 | 0 | 1 | 0 | 0 |  | Peroxidase superfamily protein |
| AT4G36520 | 0 | 0 | 0 | 0 | 0 | 0 | 1 | 0 | 0 |  | Chaperone DnaJ-domain superfamily protein |
| AT4G38050 | 0 | 0 | 0 | 1 | 1 | 0 | 0 | 1 | 1 |  | Xanthine/uracil permease family protein |
| AT4G38760 | 0 | 0 | 1 | 0 | 0 | 0 | 0 | 0 | 0 |  | nucleoporin (DUF3414) |
| AT4G39900 | 0 | 0 | 0 | 0 | 1 | 0 | 0 | 0 | 0 |  | adenine deaminase |
| AT5G01030 | 0 | 0 | 0 | 0 | 1 | 0 | 0 | 0 | 0 |  | enolase, putative (DUF3527) |
| AT5G07380 | 0 | 0 | 0 | 0 | 0 | 0 | 0 | 0 | 1 |  | hypothetical protein |
| AT5G09290 | 0 | 0 | 0 | 0 | 0 | 0 | 0 | 0 | 1 |  | Inositol monophosphatase family protein |
| AT5G10720 | 0 | 0 | 1 | 0 | 0 | 0 | 0 | 0 | 0 | *HK5* | histidine kinase 5 |
| AT5G11100 | 0 | 0 | 0 | 0 | 0 | 0 | 0 | 1 | 0 | *SYTD* | Calcium-dependent lipid-binding (CaLB domain) family protein |
| AT5G11430 | 0 | 0 | 1 | 0 | 0 | 0 | 0 | 0 | 0 |  | SPOC domain / Transcription elongation factor S-II protein |
| AT5G11490 | 0 | 0 | 0 | 0 | 0 | 0 | 0 | 0 | 1 |  | adaptin family protein |
| AT5G11530 | 0 | 0 | 0 | 0 | 0 | 0 | 1 | 0 | 0 | *EMF1* | embryonic flower 1 (EMF1) |
| AT5G12400 | 0 | 0 | 1 | 0 | 0 | 0 | 0 | 0 | 0 |  | PHD-finger and DNA binding domain-containing protein |
| AT5G13590 | 0 | 0 | 0 | 0 | 1 | 0 | 0 | 0 | 0 |  | hypothetical protein |
| AT5G13920 | 0 | 0 | 0 | 0 | 0 | 0 | 0 | 0 | 1 |  | GRF zinc finger / Zinc knuckle protein |
| AT5G14220 | 0 | 0 | 0 | 0 | 0 | 0 | 1 | 0 | 0 | *HEMG2* | Flavin containing amine oxidoreductase family |
| AT5G15270 | 0 | 0 | 1 | 0 | 0 | 0 | 0 | 0 | 0 |  | RNA-binding KH domain-containing protein |
| AT5G15380 | 0 | 0 | 0 | 0 | 0 | 0 | 1 | 0 | 1 | *DRM1* | domains rearranged methylase 1 |
| AT5G15820 | 0 | 0 | 1 | 0 | 0 | 0 | 0 | 0 | 0 |  | RING/U-box superfamily protein |
| AT5G20130 | 0 | 0 | 1 | 0 | 0 | 0 | 0 | 0 | 0 |  | sulfate adenylyltransferase subunit |
| AT5G20170 | 0 | 0 | 1 | 0 | 0 | 0 | 0 | 0 | 0 |  | RNA polymerase II transcription mediator |
| AT5G23810 | 0 | 0 | 0 | 0 | 0 | 0 | 1 | 0 | 0 | *AAP7* | amino acid permease 7 |
| AT5G24450 | 0 | 0 | 0 | 0 | 1 | 0 | 0 | 0 | 0 |  | Transcription factor IIIC, subunit 5 |
| AT5G24470 | 0 | 0 | 0 | 0 | 1 | 0 | 0 | 0 | 1 | *PRR5* | two-component response regulator-like protein |
| AT5G24500 | 0 | 0 | 0 | 0 | 0 | 0 | 0 | 0 | 1 |  | fantom protein |
| AT5G35370 | 0 | 0 | 1 | 0 | 0 | 0 | 0 | 0 | 0 |  | S-locus lectin protein kinase family protein |
| AT5G35960 | 0 | 0 | 1 | 0 | 0 | 0 | 0 | 0 | 0 |  | Protein kinase family protein |
| AT5G37510 | 0 | 0 | 0 | 0 | 0 | 0 | 0 | 0 | 1 | *EMB1467* | NADH-ubiquinone dehydrogenase |
| AT5G37830 | 0 | 0 | 0 | 0 | 0 | 0 | 0 | 0 | 1 | *OXP1* | oxoprolinase 1 |
| AT5G38840 | 0 | 0 | 0 | 0 | 0 | 0 | 1 | 0 | 0 |  | SMAD/FHA domain-containing protein |
| AT5G40480 | 0 | 0 | 0 | 0 | 0 | 0 | 0 | 0 | 1 | *EMB312* | embryo defective 3012 |
| AT5G41690 | 0 | 0 | 0 | 0 | 0 | 0 | 1 | 0 | 0 |  | RNA-binding (RRM/RBD/RNP motifs) family protein |
| AT5G43630 | 0 | 0 | 0 | 0 | 0 | 0 | 1 | 0 | 0 | *TZP* | zinc knuckle (CCHC-type) family protein |
| AT5G44800 | 0 | 0 | 0 | 0 | 0 | 0 | 1 | 0 | 1 | *CHR4* | chromatin remodeling 4 |
| AT5G45060 | 0 | 0 | 1 | 0 | 0 | 0 | 0 | 0 | 0 |  | Disease resistance protein (TIR-NBS-LRR class) family |
| AT5G45160 | 0 | 0 | 1 | 0 | 0 | 0 | 0 | 0 | 0 | *RL2* | Root hair defective 3 GTP-binding protein (RHD3) |
| AT5G45170 | 0 | 0 | 1 | 0 | 0 | 0 | 0 | 0 | 0 |  | Haloacid dehalogenase-like hydrolase (HAD) superfamily protein |
| AT5G46540 | 0 | 0 | 1 | 0 | 0 | 0 | 0 | 0 | 0 | *ABCB7* | P-glycoprotein 7 |
| AT5G54550 | 0 | 0 | 0 | 0 | 0 | 0 | 0 | 0 | 1 |  | hypothetical protein (DUF295) |
| AT5G55390 | 0 | 0 | 1 | 0 | 0 | 0 | 0 | 0 | 0 | *EDM2* | ENHANCED DOWNY MILDEW 2 |
| AT5G56900 | 0 | 0 | 1 | 0 | 0 | 0 | 0 | 0 | 0 |  | CwfJ-like family protein / zinc finger (CCCH-type) family protein |
| AT5G57770 | 0 | 0 | 1 | 0 | 0 | 0 | 0 | 0 | 0 |  | auxin canalization protein (DUF828) |
| AT5G58880 | 0 | 0 | 1 | 0 | 0 | 0 | 0 | 0 | 1 |  | LRR protein |
| AT5G60050 | 0 | 0 | 1 | 0 | 0 | 0 | 0 | 0 | 0 |  | BTB/POZ domain-containing protein |
| AT5G60060 | 0 | 0 | 1 | 0 | 0 | 0 | 0 | 0 | 0 |  | F-box SKIP23-like protein (DUF295) |
| AT5G60100 | 0 | 0 | 1 | 0 | 0 | 0 | 0 | 0 | 0 | *PRR3* | pseudo-response regulator 3 |
| AT5G60170 | 0 | 0 | 0 | 0 | 0 | 0 | 1 | 0 | 0 |  | RNA binding (RRM/RBD/RNP motifs) family protein |
| AT5G60410 | 0 | 0 | 1 | 0 | 0 | 0 | 0 | 0 | 0 | *SIZ1* | DNA-binding protein with MIZ/SP-RING zinc finger, PHD-finger and SAP domain-containing protein |
| AT5G60690 | 0 | 0 | 1 | 0 | 0 | 0 | 0 | 0 | 0 | *REV* | Homeobox-leucine zipper family protein / lipid-binding START domain-containing protein |
| AT5G61300 | 0 | 0 | 1 | 0 | 0 | 0 | 0 | 0 | 0 |  | hypothetical protein |
| AT5G61780 | 0 | 0 | 0 | 0 | 0 | 0 | 0 | 0 | 1 | *Tudor2* | TUDOR-SN protein 2 |
| AT5G62170 | 0 | 0 | 0 | 0 | 0 | 0 | 0 | 1 | 1 | *TRM25* | LOW protein: M-phase inducer phosphatase-like protein |
| AT5G62910 | 0 | 0 | 0 | 0 | 0 | 0 | 0 | 0 | 1 |  | RING/U-box superfamily protein |
| AT5G63080 | 0 | 0 | 0 | 0 | 0 | 0 | 0 | 1 | 0 |  | 2-oxoglutarate (2OG) and Fe(II)-dependent oxygenase superfamily protein |
| AT5G63120 | 0 | 0 | 0 | 1 | 0 | 0 | 0 | 1 | 0 |  | P-loop containing nucleoside triphosphate hydrolases superfamily protein |
| AT5G63320 | 0 | 0 | 0 | 0 | 0 | 0 | 0 | 0 | 1 | *NPX1* | nuclear protein X1 |
| AT5G63370 | 0 | 0 | 0 | 0 | 0 | 0 | 1 | 0 | 0 |  | Protein kinase superfamily protein |
| AT5G64010 | 0 | 0 | 1 | 0 | 0 | 0 | 0 | 0 | 0 |  | U2 small nuclear ribonucleoprotein auxiliary factor-like protein |
| AT5G65030 | 0 | 0 | 0 | 0 | 1 | 0 | 0 | 0 | 0 |  | nitric oxide synthase-interacting protein |
| AT5G65820 | 0 | 0 | 1 | 0 | 0 | 1 | 0 | 1 | 0 |  | Pentatricopeptide repeat (PPR) superfamily protein |
